# Chloride Double Perovskites Doped With Sb^3+^/Er^3+^ as Stable and Effective Luminescence Material in the Vis‐NIR Region

**DOI:** 10.1002/chem.202500066

**Published:** 2025-06-08

**Authors:** Inna A. Ivashchenko, Volodymyr V. Halyan, Lubomir D. Gulay, Volodymyr O. Yukchymchuk, Andreas Eich, Marin Rusu, Galina Gurieva, Susan Schorr, Paweł Dąbczyński, Yurij Kazarinov, Karina V. Lamonova, Oleg Khyzhun, Katarzyna Matras‐Postołek

**Affiliations:** ^1^ Cracow University of Technology Faculty of Chemical Engineering and Technology Warszawska St. 24 Cracow 31–155 Poland; ^2^ Lesya Ukrainka Volyn National University Voli Ave. 13 Lutsk 43000 Ukraine; ^3^ V.E. Lahskaryov Institute of Semiconductor Physics National Academy of Sciences Nauky ave 41 Kyiv 03028 Ukraine; ^4^ Helmholtz‐Zentrum Berlin für Materialien und Energie Hahn‐Meitner‐Platz 1 Berlin 14109 Germany; ^5^ Institut of Geological Sciences Freie Universität Berlin Kaiserswerther Str. 16–18 Berlin Germany; ^6^ Faculty of Physics Astronomy and Applied Computer Science Jagiellonian University Łojasiewicza, 11 Cracow 30–348 Poland; ^7^ NSC Kharkiv Institute of Physics and Technology Akademichna str. 1 Kharkiv 61108 Ukraine; ^8^ Max‐Born‐Institute for Nonlinear Optics and Short Pulse Spectroscopy Max‐Born‐Straße, 2A Berlin 12489 Germany; ^9^ O. O. Galkin Donetsk Institute for Physics and Engineering National Academy of Sciences of Ukraine Nauky ave. 46 Kyiv 03028 Ukraine; ^10^ Frantsevich Institute for Materials Science National Academy of Sciences of Ukraine Omeliana Pritsaka (Krzhizhanovsky) str., 3 Kyiv 03142 Ukraine; ^11^ Jan Długosz University Armii Krajowej 13/15 Czestochowa 42200 Poland

**Keywords:** doping, luminescent materials, perovskite, Vis‐NIR region

## Abstract

The luminescence properties of stable chloride double perovskites Cs_2_B^I^InCl_6_, where B^I^ is 4 at.% Ag, 6 at.% Na, doped with Sb^3+^ and Er^3+^  were investigated for the first time in the 250‐1600 nm region and revealed significant potential for advanced application. We employed XPS and ToF‐SIMS for chemical analyses, while SEM, XRD, and Raman spectroscopy provided insights into the morphology, crystal structure, and vibrational characteristics of the samples. The crystal structures of Cs_2_Ag_0.292_Na_0.708_InCl_6_, Cs_2_Ag_0.285_Na_0.715_In_0.971_Er_0.029_Cl_6_, Cs_2_Ag_0.16_Na_0.84_In_0.893_Er_0.017_Sb_0.09_Cl_6_ were examined using single‐crystal methods. The excited Sb^3^⁺ ions emitted blue light at 450 nm due to electronic absorption at sub‐band gap levels, facilitating energy transfer to Er^3^⁺ ions. Notably, the Er^3^⁺ emitted radiation at 1540 nm, a wavelength particularly useful for optical communication applications. Additionally, emissions at 525 nm, 552 nm, 665 nm, and 805 nm were observed, corresponding to *f*‐*f* transitions of Er^3^⁺ ions. These compelling results were supported with calculations based on the Modified Crystal Field Theory, explaining the effects of varying concentrations of Sb^3^⁺ and Er^3^⁺ on the crystal structure and luminescent properties. By using the synthesized materials, we successfully developed an LED prototype that utilized a UV chip (320 nm) combined with the Cs_2_Ag_0.4_Na_0.6_In_0.9_Er_0.01_Sb_0.09_Cl_6_ powder as a stable and effective luminophore for possible application in optoelectronics.

## Graphical abstract

The luminescence properties of Sb^3+^/Er^3+^‐doped chloride double perovskites Cs_2_Ag_0.4_Na_0.6_InCl_6_ have been investigated across 250‐1600 nm. Structural and spectroscopic analyses have been performed using XRD, SEM, Raman, XPS, and ToF‐SIMS methods. Energy transfer from Sb^3+^ to Er^3+^ enables emission at 525, 552, 665, 805 and 1540 nm. The LED prototype has been ‐ developed, demonstrating potential for optoelectronic applications.

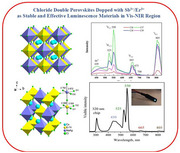

## 1 Introduction

Recently, the scientific interest in halide perovskites has been extensively rising due to their outstanding optical properties.^[^ ^1^, ^2^, ^3^ ^]^ The original mineral perovskite, CaTiO_3_, shows the general chemical composition of perovskites: ABX_3_. When A and B are fully inorganic cations, CsPbX_3_ compositions are formed, where X = Cl, Br, I.^[^ ^4^ ^]^ Finding new elements to replace Pb in the B‐site of the perovskite is essential due to its toxicity, which limits large‐scale commercial application of the lead‐perovskites. Even a low concentration of Pb can cause an environmental hazard, and it is harmful to human health as a potential carcinogen.^[^ ^5^ ^]^ As a result, the European Union has already restricted the use of toxic and heavy metals.^[^ ^6^ ^]^

**Figure 1:**
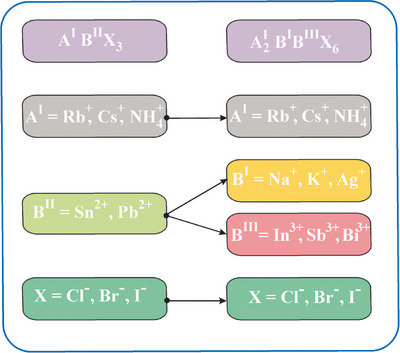
Compositions of perovskites and HDPs.

Halide elpasolites (A_2_B^I^B^III^X_6_) or halide double perovskites (HDPs), sharing a similar crystal structure with a pair of nontoxic heterovalent metal cations, are promising alternatives as they replace two toxic cations (Figure 1). An ideal lattice of HDPs is a network of corner‐sharing octahedra of heterovalent B‐site cations (e.g., Li^+^, Na^+^, K^+^, Rb^+^, Cu^+^, Ag^+^ for the B^I^ site and In^3+^, Sb^3+^, Bi^3+^, rare earth (RE) cations for the B^III^ site) with monovalent alkali cations (e.g., Cs^+^, Rb^+^, K^+^, NH_4_^+^) occupying the remaining vacancies.^[^ ^7^, ^8^, ^9^, ^10^, ^11^ ^]^

By using the ionic radii *r_A_, r_B_* = (*r*B^I^ + *r*B^III^)/2, and *r_X_*, the Goldschmidt tolerance factor *t* can be defined as *t =* (*r_A_+r_B_*)/√2(*r_B_+r_X_*). It and an octahedral factor *μ = r_B_*/*r_X_* can be used as a good screening tool to perform material design.^[^ ^12^, ^13^, ^14^ ^]^ The mild *t* variation of HDPs ensures element substitution without destroying the crystal structure, which provides many possibilities for alloying, doping, and delicate composition engineering. Experimental results demonstrate that in this case, the HDPs can, for example, transform the bandgap from indirect to direct states and significantly change the photoluminescence quantum yield (PLQY), which characterizes the photoluminescence (PL) properties (Table 1).^[^ ^10^, ^14^, ^15^, ^16^, ^17^, ^18^, ^19^ ^]^ More importantly, HDPs can emit efficient white luminescence in the form of self‐trapped excitons (STEs) (Table 1), and, consequently, can be used as cheap white phosphors.^[^ ^18^, ^20^, ^21^, ^22^ ^]^ The STEs can transfer energy to dopants, such as Mn^2+^, Cr^3+^, or RE^3+^, and further tune the emission to the near‐infrared (NIR) region.^[^ ^23^, ^24^ ^]^

**Table 1:** Optoelectronic properties of chloride DPs with luminescence from STEs.

Material	Band Gap Direct/Indirect	Emission peak, nm	PL QY %	[Ref.]
Cs_2_AgBiCl_6_	3.25/2.5	420	5	[16]
Cs_2_NaIn_0.9_Sb_0.1_Cl_6_ NCs	3.95/3.06	442	75.89	[17]
Cs_2_AgInCl_6_	3.27/‐	590	1.6	[18]
Cs_2_Ag_0.17_Na_0.83_In_0.88_Bi_0.12_Cl_6_ NCs	4.96/‐	557	64	[19]
Cs_2_Ag_0.6_Na_0.4_InCl_6_:Bi NCs	4.0/‐	558	86.2	[18]

Isovalent substitution of B^I^ and B^III^ ions is possible, and Sb^3+^, Er^3+^ alloying and doping can be a general strategy to modify the excitation of HDPs to obtain more efficient STEs or doped emission.^[^ ^24^ ^]^

The RE elements usually exist in trivalent forms with a coordination number equal to 6, making them suitable dopants for composition engineering in HDPs.^[^ ^20^ ^]^ As previously mentioned, Cs_2_Na(Ag)InCl_6_, Cs_2_Na(Ag)SbCl_6_, and Cs_2_Na(Ag)RECl_6_ crystallize in cubic symmetry, Sp.Gr. $\mathrm{Fm}\bar{3}m$.^[^ ^10^, ^12^ ^]^ Based on these considerations, many multimode‐excited luminescent materials have been developed.^[^ ^24^, ^25^, ^26^, ^27^, ^28^ ^]^

A very important question is to choose the most optimal compositions of the HDPs matrix for the following alloying and doping. Cs_2_AgInCl_6_ has been investigated in some publications, but it has weak PL. An enhancement of the PLQY can be achieved by introducing Na^+^ to break the parity‐forbidden transition in Cs_2_AgInCl_6_.^[^ ^9^, ^10^, ^18^ ^]^ Because of a Jahn‐Teller distortion of the [AgCl_6_]^5‐^ octahedra in this case, STEs appear in the excited state, resulting in the broadband emission of Cs_2_Ag_1‐_ *_x_* Na*_x_* InCl_6_.^[^ ^9^, ^11^, ^18^, ^21^ ^]^ The most optimal composition could be Cs_2_Ag_0.4_Na_0.6_InCl_6_ to obtain the highest PLQY.^18^, ^21^ ^]^ One way to optimize optical properties is to dope the samples with *p*‐elements, like Sb^3+^ or Bi^3+^.^[^ ^9^, ^10^, ^14^, ^16^, ^17^, ^19^, ^20^, ^21^ ^]^ In this way, it is possible to highly increase the PLQY, even up to 86%.^[^ ^9^, ^10^, ^18^ ^]^

Stability is a challenging issue for the commercial application of halide perovskites, for example, CsPbX_3_ have poor light, heat, and moisture stability, while chloride DPs, for example, Cs_2_NaInCl_6_, show excellent environmental stability.^[^ ^17^ ^]^ Moreover, Cs_2_NaInCl_6_:Sb^3+^ decomposes at 870 K, that is, at a higher temperature than Cs_2_AgInCl_6_ (780 K) and Cs_2_AgSbCl_6_ (520 K).

There are two mostly usable synthesis methods of HDP powder samples, namely, hydrothermal and precipitation techniques.^[^ ^10^, ^24^, ^28^ ^]^ Their main disadvantage is the usage of 36% HCl acid, difficulties in the composition homogeneity of the polycrystals, and the tiny crystal size, which makes it challenging to investigate them by (single crystal) X‐ray diffraction methods. Solid‐state reaction^[^ ^10^, ^11^, ^21^ ^]^ is less used compared with hydrothermal and precipitation methods. Nevertheless, in this way, it is possible to obtain alloys with uniformly distributed elements, which is crucial for composition engineering. Moreover, this reaction can provide suitable samples for crystal structure investigation by X‐ray single crystal diffraction methods.

Taking the above‐mentioned issues into consideration, the main aims of the presented research are: (i) using the solid‐state technique, through careful composition engineering with Sb^3+^, Er^3+^, to prepare stable, efficient photoluminescent chloride DP samples based on Cs_2_Ag_0.4_Na_0.6_InCl_6_; (ii) to study the optoelectronic properties of the obtained microcrystal samples of the chloride DPs; (iii) to find the possibility of covering the visible‐near‐infrared (Vis‐NIR) range based on the relationship between composition, structure, and properties of the chloride DPs.

## 2 Results and Discussion

### 2.1 Results of XRD Studies

All the synthesized chloride DPs belong to the $Fm\bar{3}m$ space group with the lattice parameters: *a* = 10.4806(2) Å for Cs_2_AgInCl_6_, *a* = 10.5114(2) Å for Cs_2_Ag_0.4_Na_0.6_InCl_6_, and *a* = 10.5359(2) Å for the Cs_2_NaInCl_6_ sample (Figure S1, Table S1, S2, S3, S4), which agrees well with other findings.^[^ ^8^, ^17^, ^19^, ^21^ ^]^ By comparing the diffractograms, it is evident that the intensity of the 111 Bragg peak changes as the amount of sodium enlarges from 0 to 1. This fact confirms the incorporation of Na^+^ and Ag^+^, into the B^I^ site of the crystal structure of Cs_2_B^I^InCl_6_ chloride DPs.

For the doped samples Cl6, Cl7, and Cl8, the crystal structure refinement was also performed based on the powder XRD patterns (Figure S2, Table S1, S5–S7). Doping with Er^3+^ and Sb^3+^ increases the lattice parameters of the unit cell (Table S1) and agrees with literature.^[^ ^19^, ^20^, ^21^ ^]^ The dopants are homogeneously distributed in the samples (Figures S3, S4, Table S8). Since the concentrations of Sb^3+^ and Er^3+^ dopants are low, their quantitative estimation from EDS is less reliable.

**Figure 2:**
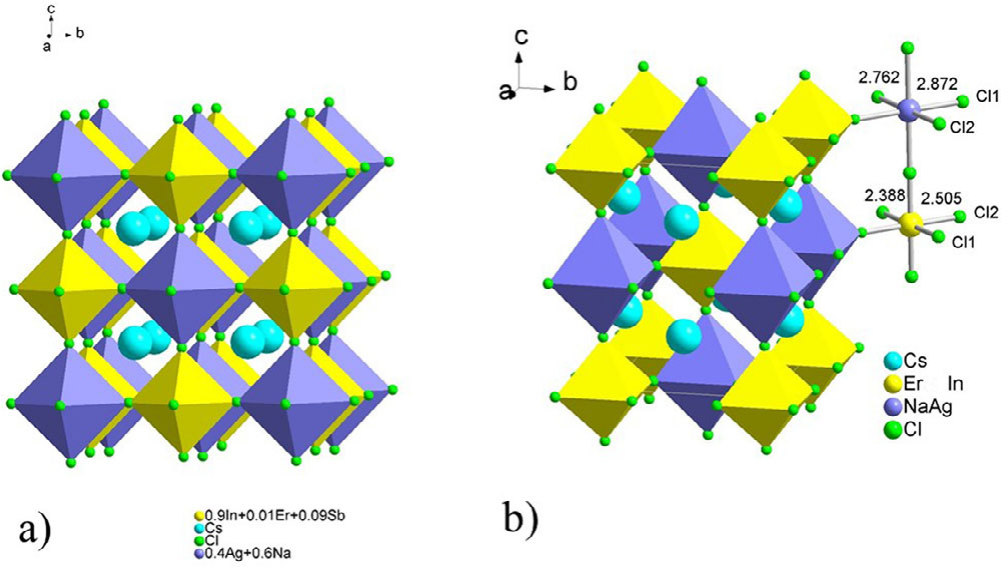
a) The cubic structure of the lead‐free double perovskite Cs_2_Ag_0.4_Na_0.6_In_0.9_Er_0.01_Sb_0.09_Cl_6_ (Cl7); b) tetragonal crystal structure of the Cs_2_Ag_0.4_Na_0.6_In_0.9_Er_0.1_Cl_6_ (Cl6) chloride DPs.

The cubic unit cell framework of the Cl7 sample is presented in Figure 2a and is constructed with [M_1_Cl_6_] and [M_2_Cl_6_] octahedra, where M_1_ = 40% Ag + 60% Na, M_2_ = 90% In + 1% Er + 9% Sb. The expected lead‐free double perovskite structure of Cs_2_Ag_0.4_Na_0.6_In_0.9_Sb_0.09_Er_0.01_Cl_6_ (Cl7) is a network of corner‐sharing octahedra of M_1_ mixture (light violet octahedra) for the B^I^ site and M_2_ mixture for the B^III^ site (yellow octahedra), with monovalent Cs cations occupying the rest of the cavities (blue balls). The Rietveld refinement of the crystal structures of Cl6, Cl8 showed a tetragonal syngony with the I4/mmm space group (Figure 2b, Table S1, S5–S7).

To verify the powder XRD‐based crystal structure refinement of Cs_2_Ag_0.4_Na_0.6_InCl_6_, Cs_2_Ag_0.4_Na_0.6_In_0.9_Er_0.1_Cl_6_ (Cl6), and Cs_2_Ag_0.4_Na_0.6_In_0.9_Sb_0.09_Er_0.01_Cl_6_ (Cl7) samples, single crystals suitable for further study were selected from them, microscopically examined, and characterised (Tables S9–S11). The analysis of the *hkl* indexes of the reflexes indicated a cubic syngony with $Fm\bar{3}m$ space group, in which the refinement of the structures was performed. The calculated interatomic distances and coordination numbers of the atoms in the structures are given in Table S12. The distances agree well with the sum of the radii of the corresponding ions (Ag^+^: 115 pm; Na^+^: 102 pm; In^3+^: 80 pm; Er^3+^: 89 pm; Sb^3+^: 76 pm).^[^ ^29^ ^]^

The difference in the crystal structure for the Cl6 sample based on powder XRD and single crystal investigations (Table S1) could be explained by the difference in the composition of the powder sample, obtained from the total alloy of the Cl6 sample (Table S8, Figure S3). The tiny crystal used to study the crystal structure has a lower Er content, because it was selected from the upper part of the sample, where the best‐shaped crystals had been found after synthesis in the quartz ampoules (Table S13). The larger ionic size of Er^3+^ could be a possible reason for the lower amount of Er in the crystals. The Sb^3+^ ions have a higher tendency to incorporate the structure of the chloride DPs due to the similar size of Sb^3+^ and In^3+^ in their octahedral coordination.

### 2.2 EDS and ToF‐SIMS Results

**Figure 3:**
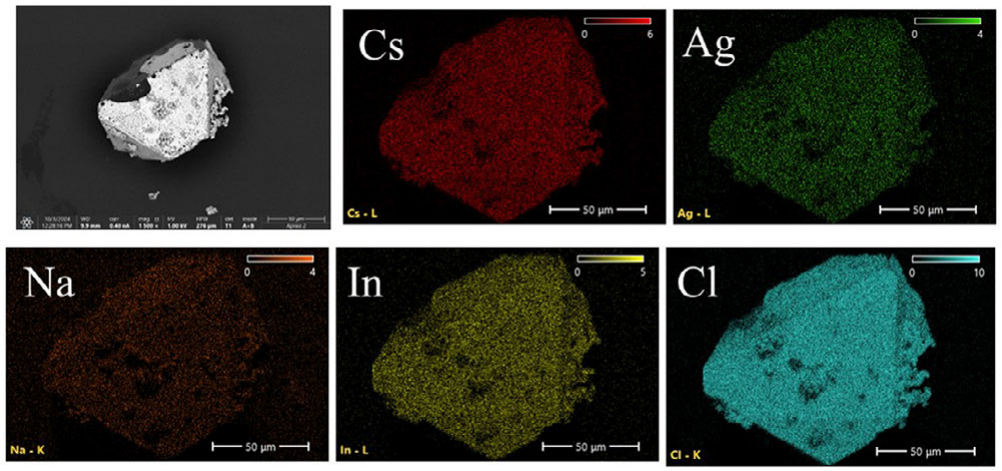
Maps of the elements of the single crystal Cs_2_Ag_0.292_Na_0.708_InCl_6_ (Cs_2_Ag_0.4_Na_0.6_InCl_6_ batch composition).

**Figure 4:**
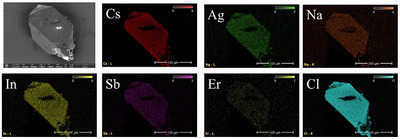
Maps of the elements of the Cl7 single crystal Cs_2_Ag_0.160_Na_0.840_In_0.893_Er_0.017_Sb_0.090_Cl_6_ (Cs_2_Ag_0.4_Na_0.6_In_0.9_Er_0.01_Sb_0.09_Cl_6_ batch composition).

The elemental ratio obtained from the EDS analysis (Table S13) supports the crystal structure determination of the chloride DPs (Tables S9–S12). The elemental mapping data show that the dopants are homogeneously distributed (Figures 3, 4, S5), but it should be noted that the amounts of Er are lower than calculated (Table S13), which can be explained by the difficulties in Er incorporation into the crystal structure of the chloride DPs.

**Figure 5:**
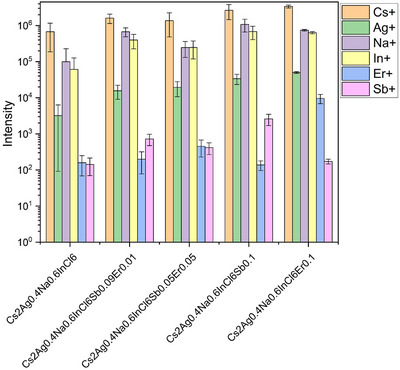
Peak area statistics of chosen elements from the mass spectra collected for the powder samples.

According to the ToF‐SIMS results, for all the samples the Cs, Ag, Na, and In signals were clearly detected in the mass spectra (Figure 5, S6, S7, S8). Also, where Er and/or Sb were added to the system, the masses of the respective elements can also be detected. As can be seen by the analysis of the peak area, all samples follow the expected trend. However, it is worth noting that a sample in the powder form is characterized by macroscopic roughness. Consequently, the values of the peak area can be significantly different from point‐to‐point and should not be treated as an absolute quantity, but we can use it to follow the sample‐to‐sample trends.

### 2.3 Raman Spectra Description

**Figure 6:**
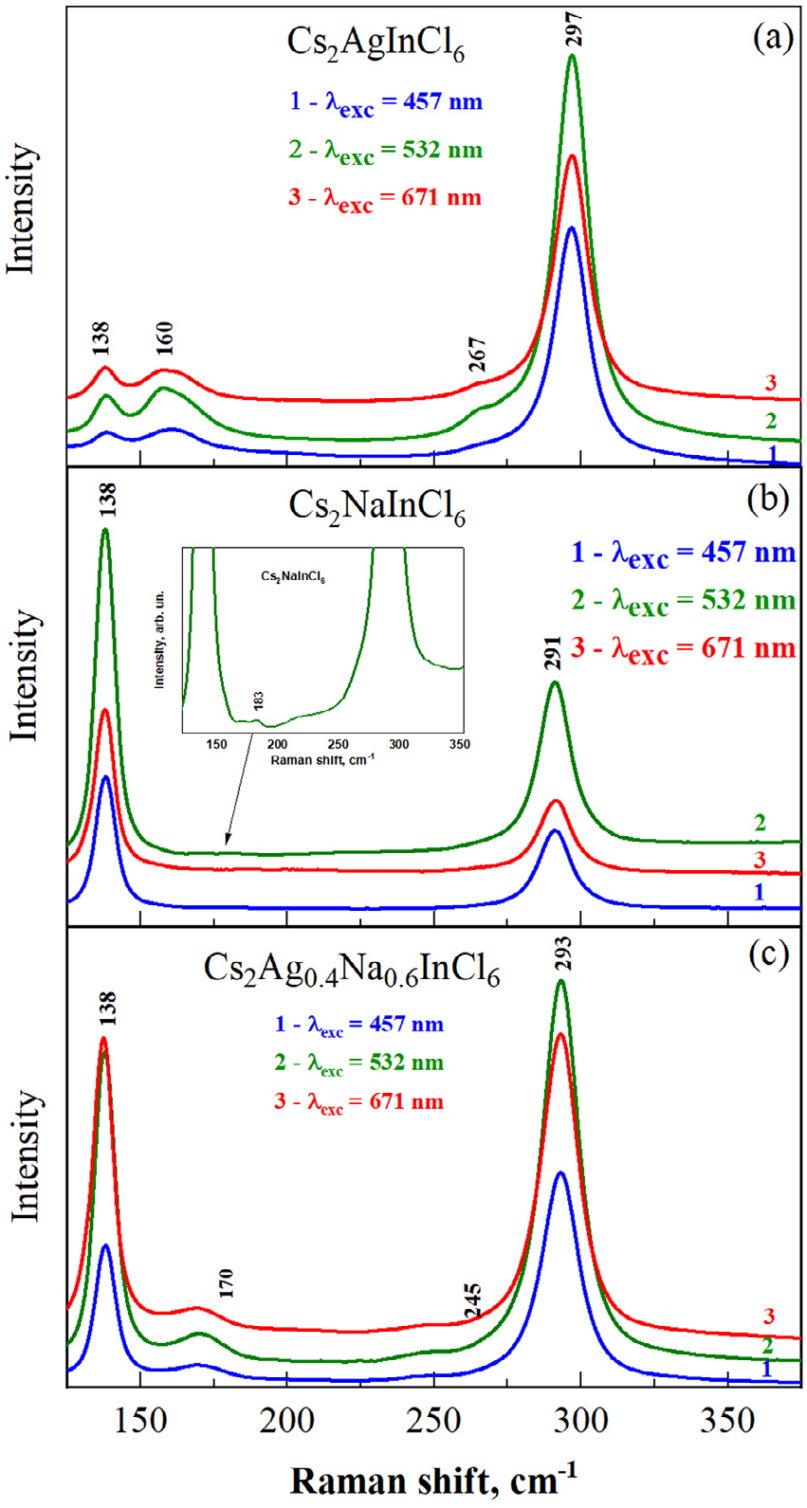
Raman spectra of Cs_2_AgInCl_6_ a), Cs_2_NaInCl_6_ b), and Cs_2_Ag_0.4_Na_0.6_InCl_6_ c) at 457, 532, and 671 nm laser radiation.

Raman spectra of Cs_2_AgInCl_6_ (Figure 6a), Cs_2_NaInCl_6_ (Figure 6b), and Cs_2_Ag_0.4_Na_0.6_InCl_6_ (Figure 6c) were obtained using laser radiation with different wavelengths of 457, 532, and 671 nm. According to the selection rules for double perovskites of type A_2_B^I^B^III^X_6_, the Raman spectra exhibit four vibrational modes: *A*_1g_, *E*_g_, and 2*F*_2g_.^[^ ^30^ ^]^ One of the (*F*_2g_) modes with a low frequency $F_{2g}^{L}$ is assigned to the translational lattice motion of Cs^+^ ions in the octahedral space. Another *F*_2g_ mode, with a high frequency $F_{2g}^{H}$, refers to bending vibrations of the octahedral cages.^[^ ^31^ ^]^ The *A*_1g_ vibrational mode refers to the symmetric stretching mode, and *E*_g_ refers to the antisymmetric stretching mode. As can be seen from Figure 6a–c, the wavelength variation of the exciting laser radiation has almost no effect on the change in the spectra, indicating the absence of resonance effects in this energy range (2.71–1.85 eV).

**Figure 7:**
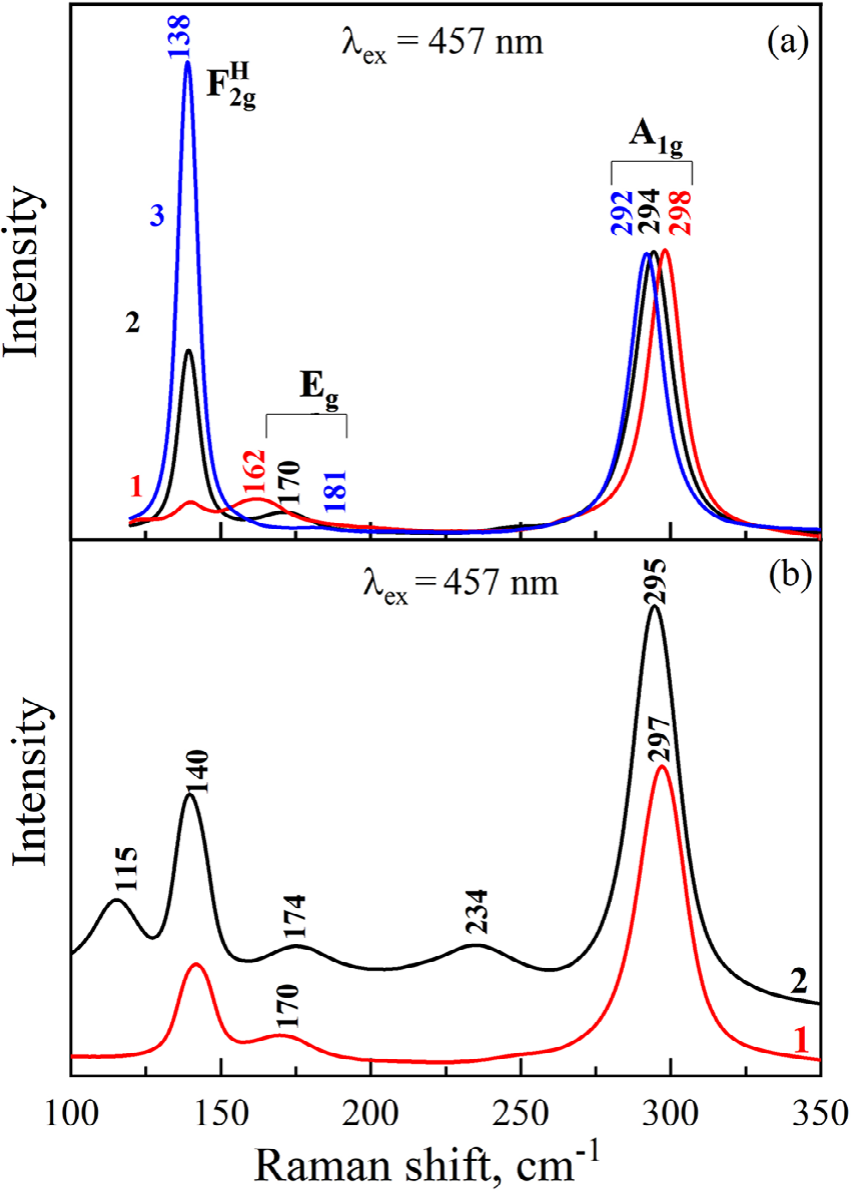
The normalized Raman spectra of Cs_2_AgInCl_6_ (curve 1), Cs_2_Ag_0.4_Na_0.6_InCl_6_ (curve 2), and Cs_2_NaInCl_6_ (curve 3), obtained at 457 nm laser radiation a); Raman spectra of Cs_2_Ag_0.4_Na_0.6_In_0.9_Er_0.1_Cl_6_ (Cl6) (curve 1) and Cs_2_Ag_0.4_Na_0.6_In_0.9_Er_0.05_Sb_0.05_Cl_6_ (Cl8) (curve 2) excited by 457 nm laser radiation.

Figure 7a shows the Raman spectra of Cs_2_NaInCl_6_, Cs_2_Ag_0.4_Na_0.6_InCl_6_, and Cs_2_AgInCl_6_ samples normalized to the intensity of the *A*_1g_ band. As can be seen in this figure, the frequency positions of the *A*_1g_ vibrational mode of these compounds are quite close and equal 292, 294, and 298 cm^−1^, respectively. This is explained by the fact that Ag, Na, and In ions, which are located in the centers of the octahedra, practically do not oscillate (Tables S2–S4). This replacement of the heavy Ag ion with lighter Na practically does not affect the frequency of the *A*_1g_ vibrational mode. At the same time, the difference in the ionic radii of Ag^+^ (115 pm) and Na^+^ (102 pm) and, accordingly, the different lengths of Ag─Cl and Na─Cl bonds^[^ ^29^ ^]^ cause such force constants, which determine the frequency of the *A*_1g_ vibrational mode. In Cs_2_Ag_0.4_Na_0.6_InCl_6_ with almost the same number of Ag and Na atoms, the frequency position of the *A*_1g_ band is intermediate between Cs_2_AgInCl_6_ and Cs_2_NaInCl_6_. In the case of the antisymmetric stretching mode *E*_g_, its frequency position, in addition to the force constants and mass of Cl atoms, is also affected by the parameter that quantifies the relative strength of the repulsive interaction generated by two neighboring Cl atoms concerning their equilibrium separation,^[^ ^32^ ^]^ which is different for these compounds. Therefore, the *E*_g_ mode of Cs_2_AgInCl_6_ has a much lower frequency (162 cm^−1^) compared to the mode frequency of Cs_2_NaInCl_6_ (181 cm^−1^).

The positions of the vibration modes $F_{2g}^{H}$ for different double perovskites are equal to 138 cm^−1^, while their intensities differ significantly. Since a similar ratio of the mode intensities is observed when Raman spectra are excited with wavelengths of 457, 532, and 671 nm, the influence of resonance effects on the mode intensities can be disregarded. Therefore, the oscillator strengths for the vibrational modes of different perovskites are likely to be very different.

**Figure 8:**
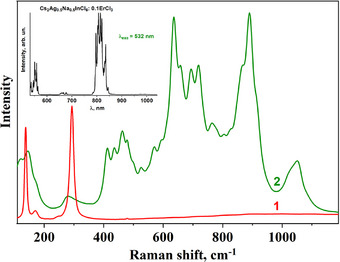
Raman spectra of Cs_2_Ag_0.4_Na_0.6_InCl_6_ (curve 1) and Cs_2_Ag_0.4_Na_0.6_In_0.9_Er_0.1_Cl_6_ (Cl6) (curve 2) excited with 532 nm laser radiation.

**Figure 9:**
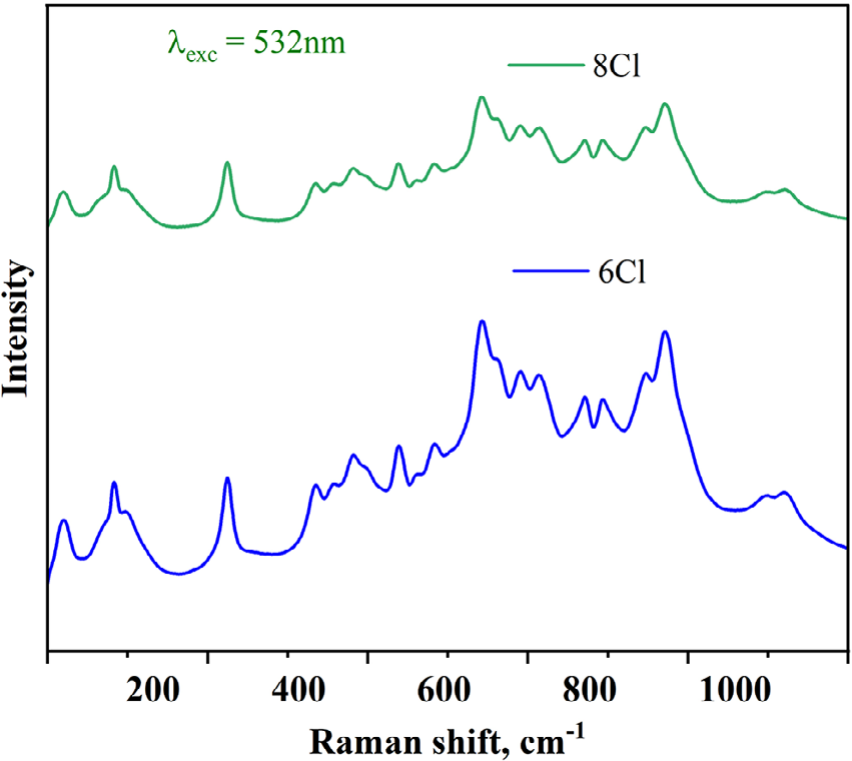
Raman spectra of Cs_2_Ag_0.4_Na_0.6_In_0.9_Er_0.1_Cl_6_ (Cl6) and Cs_2_Ag_0.4_Na_0.6_In_0.9_Er_0.05_Sb_0.05_Cl_6_ (Cl8) excited with 532 nm laser radiation.

Figure 7b shows Raman spectra of Cs_2_Ag_0.4_Na_0.6_In_0.9_Er_0.1_Cl_6_ (Cl6) and Cs_2_Ag_0.4_Na_0.6_In_0.9_Er_0.05_Sb_0.05_Cl_6_ (Cl8) samples excited with 457 nm laser radiation. There are modes (115 cm^−1^, 234 cm^−1^) that can be explained with Sb^3+^ incorporation into the crystal structure. Figure 8 shows Raman spectra of Cs_2_Ag_0.4_Na_0.6_InCl_6_ (curve 1) and Cs_2_Ag_0.4_Na_0.6_In_0.9_Er_0.1_Cl_6_ (Cl6) (curve 2) excited with 532 nm laser radiation. The spectrum of the Er‐doped double perovskite differs significantly from the undoped sample. All modes in the 400─1200 cm^−1^ region are caused by radiative transitions of electrons in the *f*‐shell of Er^3+^. The incorporation of cations into the Cs_2_Ag_0.4_Na_0.6_InCl_6_ structure leads to the splitting of the Er terms by the crystal field even at room temperature.^[^ ^33^ ^]^ The measurement of the spectrum in a wider spectral range shows that it is PL of Er (see inset to Figure 8). This conclusion can be supported by the fact that the same spectra were obtained for other Er^3+^‐doped Cl6 and Cl8 samples (Figure 9).

The obtained Raman modes, therefore, provide evidence for the doping of Sb^3+^ and Er^3+^ in the host structure (Cs_2_Ag_0.4_Na_0.6_InCl_6_) with respective effects on the normal modes of vibration.

### 2.4 XPS Result Characterization

Results of XPS measurement for Cs_2_Ag_0.4_Na_0.6_InCl_6_, Cs_2_Ag_0.4_Na_0.6_In_0.9_Sb_0.1_Cl_6_ (Cl6), Cs_2_Ag_0.4_Na_0.6_In_0.9_Sb_0.09_Er_0.01_Cl_6_ (Cl7), and Cs_2_Ag_0.4_Na_0.6_In_0.9_Sb_0.05_Er_0.05_Cl_6_ (Cl8) samples indicate that all the spectral peculiarities can be attributed to those associated with elements composing the samples under consideration (Figure S9).

In addition, XPS spectra associated with oxygen and carbon were also detected, which could be described by oxygen‐ and carbon‐bearing species adsorbed on the sample surfaces because of contact with laboratory air for a rather long time prior to the present XPS measurements. However, the relative intensities of the spectral peculiarities are not high. Figure S10 presents results of detailed studies of the most informative XPS core‐level spectra associated with caesium, chlorine, silver, and indium atoms (the Cs 3*d*, Cl 2*p*, Ag 3*d*, and In 3*d* spectra, respectively). They indicate that the charge states of Cs in the studied compounds are close to Cs^+^, while those of Ag and In atoms are somewhat smaller than expected to be for the cations of the chemical elements.^[^ ^34^ ^]^ Similarly to the XPS studies of the related Cs‐containing halide perovskites,^[^ ^35^ ^]^ this suggests a significant covalent component of the chemical bonding in addition to the ionic component. Furthermore, a comparison of the binding energies of the Cs 3*d*_5/2_ and Cl 2*p* core‐level spectra (Figure S10, a–d) indicates that the ionic degree of the Cs─Cl bonds is close to that parameter in perovskite CsPbCl_3_.^[^ ^35^ ^]^ As can be seen from deconvolutions of the XPS core‐level spectra presented in Figure S10, the Cs atoms, in addition to Cs^+^ charge, reveal some small amounts of Cs^0^. We did not detect essential changes of the charge states of the Cs, Cl, Ag, and In atoms in Cs_2_Ag_0.4_Na_0.6_InCl_6_, Cs_2_Ag_0.4_Na_0.6_In_0.9_Sb_0.1_Cl_6_, and Er‐doped Cs_2_Ag_0.4_Na_0.6_In_0.9_Sb_0.09_Er_0.01_Cl_6_, Cs_2_Ag_0.4_Na_0.6_In_0.9_Sb_0.05_Er_0.05_Cl_6_ samples because of no substantial shifts of the XPS core‐level Cs 3*d*, Cl 2*p*, Ag 3*d*, and In 3*d* spectra (Figure S10). It is worth mentioning that the most informative spectra associated with sodium (Na 1*s*), antimony (Sb 3*d*_5/2_), and erbium (Er 4*d*) superimpose more intensive Auger in M_45_N_45_N_45_ lines, and core‐level O 1*s* and Cs 4*p*_1/2_ lines, respectively (Figure S9). This fact did not allow us to estimate precisely the chemical compositions of the investigated samples, also because of a very small amount of the additives.

### 2.5 Visible and NIR Emission

**Figure 10:**
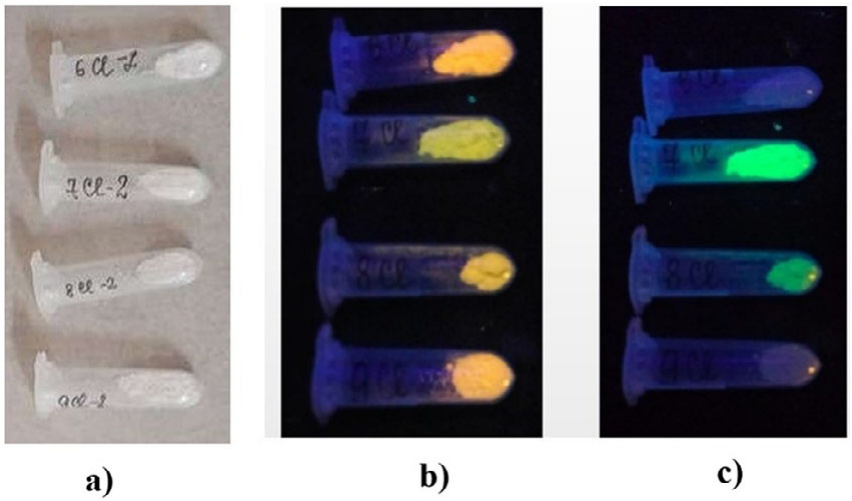
Obtained samples Cl6, Cl7, Cl8, Cl9 studied under the light with different wavelengths: a) under daylight; b) under 254 nm; c) under 365 nm.

**Figure 11:**
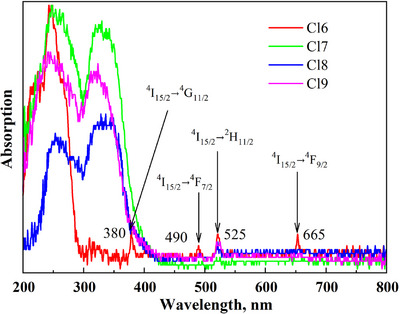
Absorption spectra of the Cs_2_Ag_0.4_Na_0.6_In_0.9_Er_0.1_Cl_6_ (Cl6), Cs_2_Ag_0.4_Na_0.6_In_0.9_Er_0.01_Sb_0.09_Cl_6_ (Cl7), Cs_2_Ag_0.4_Na_0.6_In_0.9_Er_0.05_Sb_0.05_Cl_6_ (Cl8), and Cs_2_Ag_0.4_Na_0.6_In_0.9_Er_0.07_Sb_0.03_Cl_6_ (Cl9) samples measured in the 200–800 nm range.

The obtained samples (Figure 10a) revealed bright luminescence in the visible range (Figure 10b, c). The optical absorption spectra of the Cl6, Cl7, Cl8, and Cl9 samples were measured in the 200–800 nm range (Figure 11). Notably, the Cl6 sample has an absorption edge in the ultraviolet range around 300 nm. The addition of the Sb^3+^ in Cl7 causes a significant shift (∼100 nm) toward longer wavelengths. Literature data show that the ground state of Sb^3+^ is ^1^S_0_ (5s^2^) and the excited states ^3^P_0_, ^3^P_1_^*^, ^3^P_2_, and ^1^P_1_^*^ correspond to 5s^1^p^1^ configuration. The absorption bands above 260 nm are attributed to the 5*s* ^2^ →5*s* ^1^5*p* ^1^ transition.^[^ ^36^, ^37^ ^]^ Moreover, it has been determined that for an Sb^3+^ ion, the spin‐orbit interaction is significant ($\xi_{Sb^{3+}}=4800$ cm^−1^) and ^1^P_1_^*^, ^3^P_1_^*^ excited terms influence each other.^[^ ^38^ ^]^ Although the transitions from ^1^S_0_ to ^3^P_0_, ^3^P_1_, and ^3^P_2_ are spin‐forbidden, the ^1^S_0_ → ^3^P_1_^*^ and ^1^S_0_ → ^3^P_2_^*^ transitions become partially allowed because of spin‐orbit coupling and lattice vibrations. The ^1^S_0_ → ^3^P_1_^*^ is the A‐band with the absorption at ∼320 nm, the ^1^S_0_ → ^3^P_2_^*^ is the B‐band with the absorption at ∼270 nm, and ^1^S_0_ → ^1^P_1_^*^ is the C‐band (a spin‐allowed transition) with the absorption at ∼250 nm. ^[^ ^37^ ^]^

In addition, the narrow absorption bands with maxima at 380, 490, 525, and 665 nm were recorded and associated with transitions ^4^I_15/2_ → ^4^G_11/2_, ^4^I_15/2_ → ^4^F_7/2_, ^4^I_15/2_ → ^2^H_11/2_, and ^4^I_15/2_ → ^4^F_9/2_ in the *f*‐shell of Er^3+^ ions. When the Er content increases (samples Cl8, Cl9), the reverse process occurs, like a slight shift of the absorption edge toward the ultraviolet side (∼380 nm).

**Figure 12:**
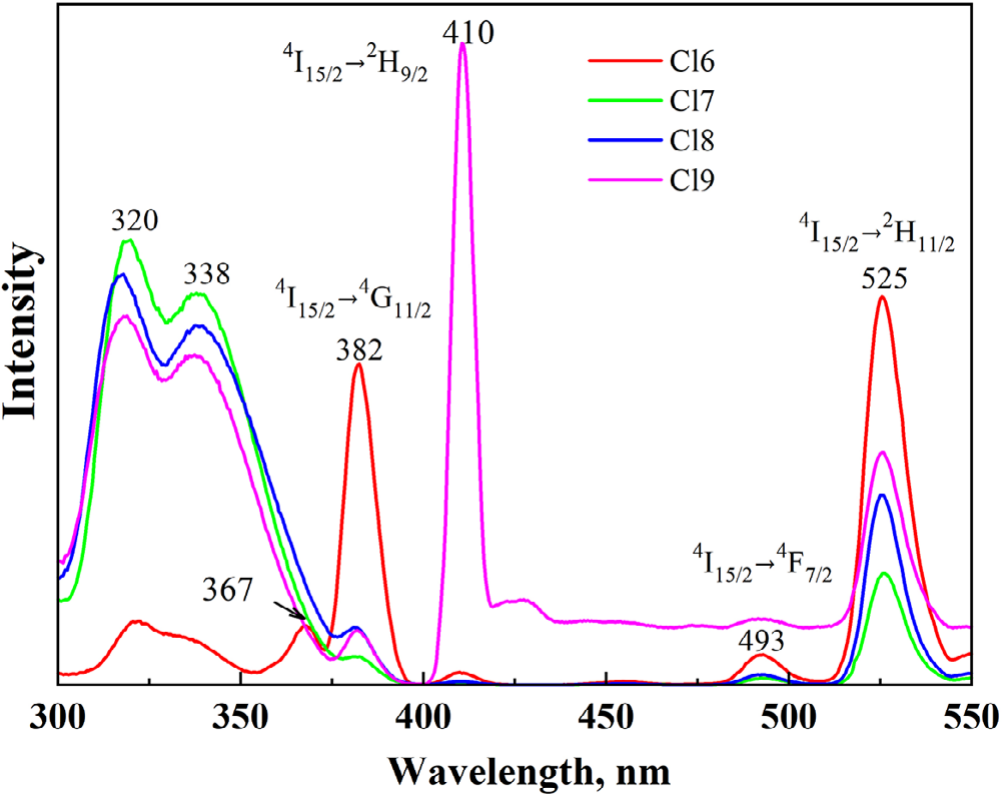
PLE spectra at the emission wavelengths 805 nm for Er^3+^‐doped Cs_2_Ag_0.4_Na_0.6_In_0.9_Er_0.1_Cl_6_ (Cl6) and Sb^3+^–Er^3+^ co‐doped samples Cs_2_Ag_0.4_Na_0.6_In_0.9_Er_0.01_Sb_0.09_Cl_6_ (Cl7), Cs_2_Ag_0.4_Na_0.6_In_0.9_Er_0.05_Sb_0.05_Cl_6_ (Cl8), and Cs_2_Ag_0.4_Na_0.6_In_0.9_Er_0.07_Sb_0.03_Cl_6_ (Cl9).

PL excitation (PLE) spectra were collected for both Er^3+^‐doped (Cl6) and Sb^3+^–Er^3+^ co‐doped Cs_2_Ag_0.4_Na_0.6_InCl_6_ (Cl7, Cl8, Cl9) samples (Figure 12). There are two bands at 320 nm and 338 nm because of the excitation transitions in Sb^3+^, which agrees well with literature.^[^ ^37^, ^39^ ^]^ Moreover, there are narrow bands at 380 nm, 410 nm, 493 nm, and 525 nm because of the excitation of Er^3+^.

The possible energy transfer between chloride DP crystals and Er ions and the effect of this process on the mechanism and efficiency of radiation is an interesting issue. To solve this, we investigated the PL properties of the samples using two excitation wavelengths: 320 nm, which is the most effective wavelength to excite these double perovskites (Figure 12), and 805 nm, which is the excitation wavelength of Er ions.

**Figure 13:**
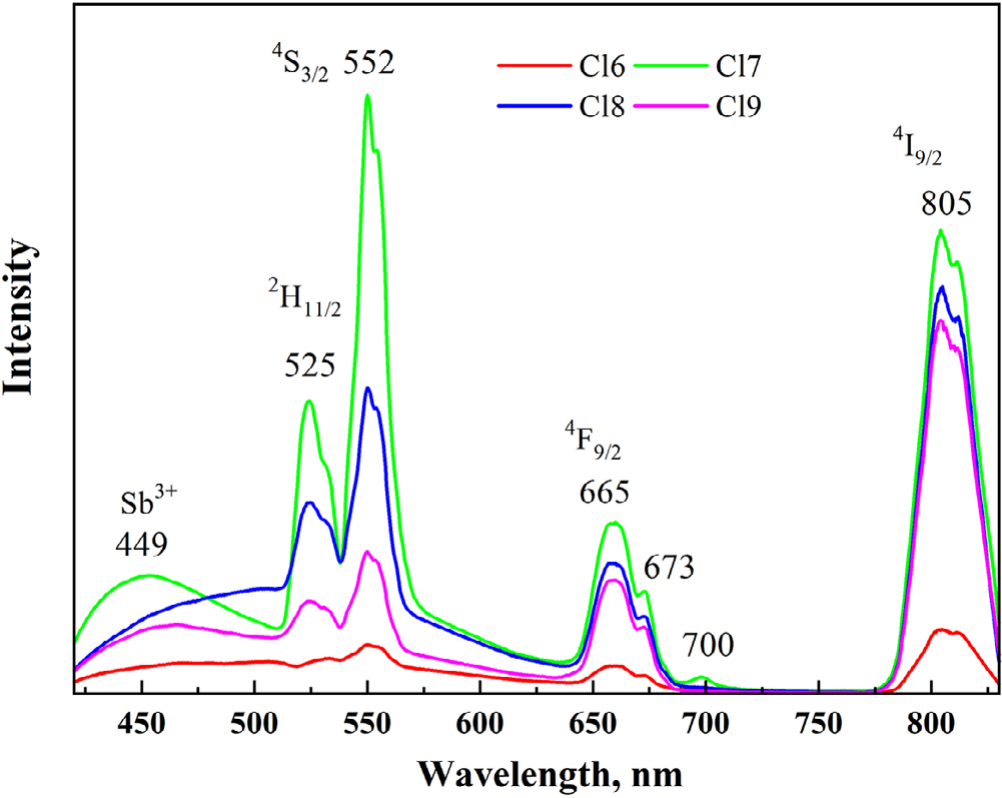
The emission spectra of the synthesized samples Cs_2_Ag_0.4_Na_0.6_In_0.9_Er_0.1_Cl_6_ (Cl6), Cs_2_Ag_0.4_Na_0.6_In_0.9_Er_0.01_Sb_0.09_Cl_6_ (Cl7), Cs_2_Ag_0.4_Na_0.6_In_0.9_Er_0.05_Sb_0.05_Cl_6_ (Cl8), and Cs_2_Ag_0.4_Na_0.6_In_0.9_Er_0.07_Sb_0.03_Cl_6_ (Cl9) obtained under 320 nm wavelength.

The emission spectra of chloride DPs obtained under 320 nm wavelength are shown in Figure 13. According to the literature,^[^ ^19^, ^21^ ^]^ a broad band of 420–620 nm corresponds to the emission of chloride DPs. In addition, the intense PL bands with maxima at 525, 552, 665, and 805 nm correspond to transitions ^2^H_11/2_ → ^4^I_15/2_, ^4^S_3/2_ → ^4^I_15/2_, ^4^F_9/2_ → ^4^I_15/2_, ^4^I_9/2_ → ^4^I_15/2_ in the *f*‐shell of Er^3+^ ions. The “blue” PL band is obtained due to the emission of Sb^3+^‐doped chloride DPs (with the maximum at 450 nm), and the “green” and “red” ones are due to the emission of Er^3+^.

**Figure 14:**
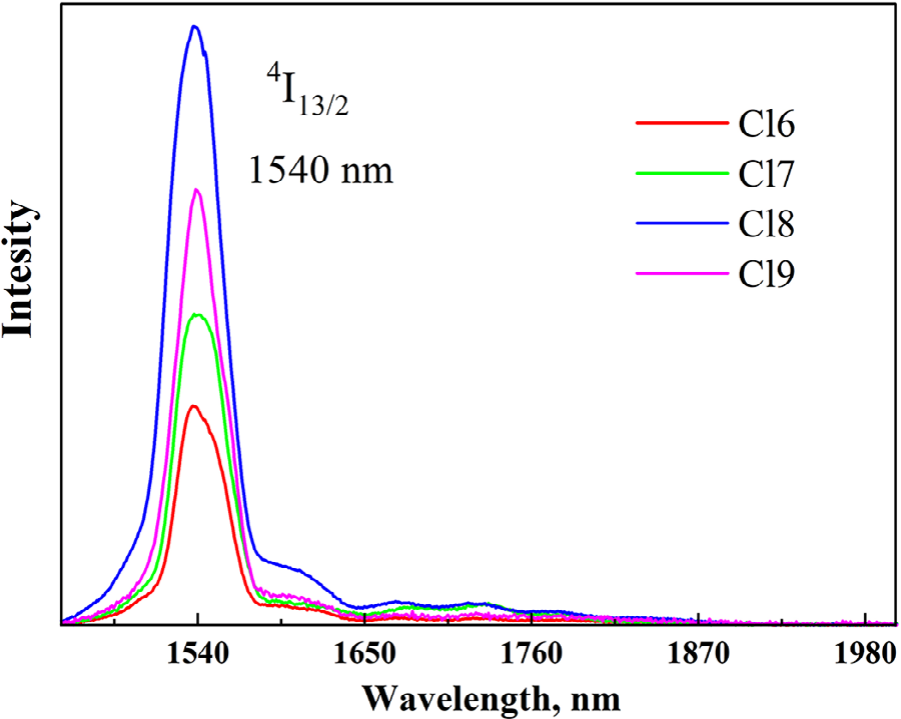
The Stokes PL spectra of the Cs_2_Ag_0.4_Na_0.6_In_0.9_Er_0.1_Cl_6_ (Cl6), Cs_2_Ag_0.4_Na_0.6_In_0.9_Er_0.01_Sb_0.09_Cl_6_ (Cl7), Cs_2_Ag_0.4_Na_0.6_In_0.9_Er_0.05_Sb_0.05_Cl_6_ (Cl8), and Cs_2_Ag_0.4_Na_0.6_In_0.9_Er_0.07_Sb_0.03_Cl_6_ (Cl9) samples under 805 nm excitation.

The down‐conversion PL spectra of the Er^3+^‐doped samples under 805 nm excitation were measured (Figure 14). The most intense PL band was observed in the NIR range with a peak at 1540 nm, corresponding to the ^4^I_13/2_ → ^4^I_15/2_ transitions in Er^3+^ ions. It should be noted that the band intensity does not follow a simple trend with the Er concentration. It increases in the Cl7 (0.1 at.% Er) and Cl8 (0.5 at.% Er) samples and then decreases sharply in Cl9 (0.7 at.% Er) and Cl6 (1 at.% Er). This is due to the phenomenon of concentration quenching, which results from an increase in the probability of energy transfer between Er^3+^ ions and nonradiative transitions. The same concentration dependence of the PL intensity was consistent in the PL spectra when excited with 320 nm (Figure 13). The same applies to the Sb concentration dependence. The intensity of the 1540 nm band increases linearly in the Cl6 (0 at.% Sb), Cl9 (0.3 at.% Sb), and Cl8 (0.5 at.% Sb) samples and then decreases in Cl7 (0.9 at.% Sb). Therefore, the best result was achieved in the Cl8 sample with 0.5 at.% Er and 0.5% Sb for the emission in the NIR range.

**Figure 15:**
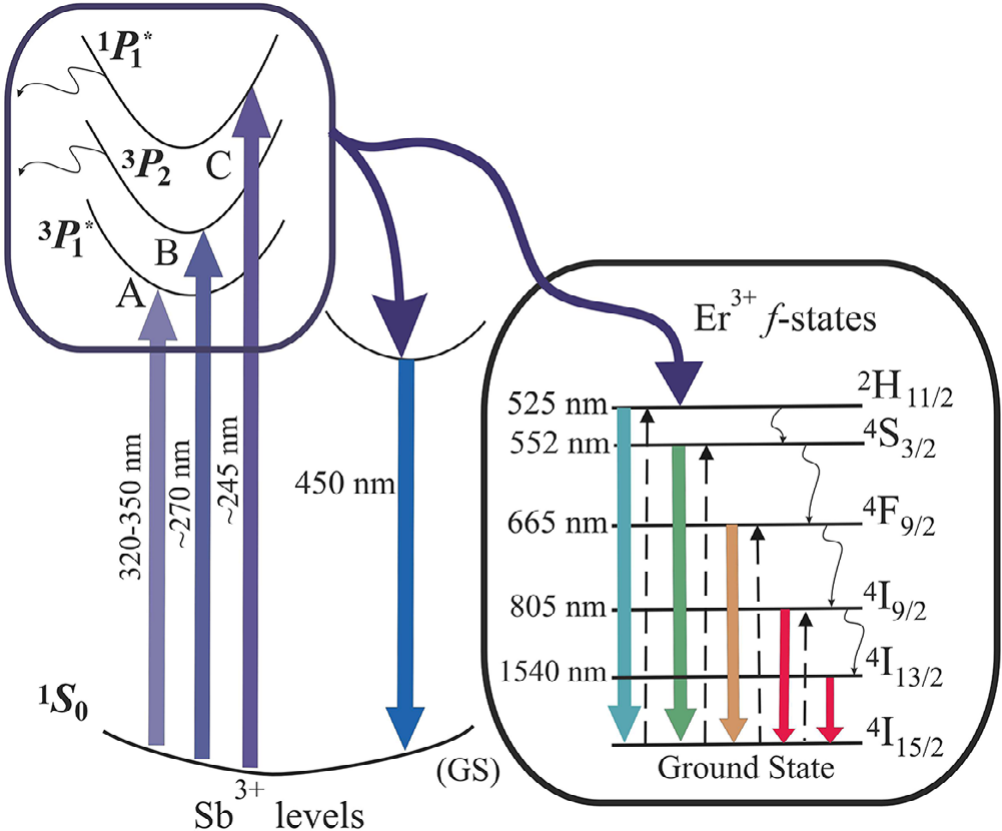
A possible emission mechanism in the Sb^3+^ and Er^3+^‐doped samples.

Based on literature data,^[^ ^40^ ^]^ the emission mechanism can be explained as energy absorption of Sb^3+^ at ∼330 nm (A‐band), 270 nm (B‐band), and 245 nm (C‐band), with the following strong emissions at 450 nm and simultaneous emission of Er ions at Vis‐ and NIR regions: (525 nm) ^2^H_11/2_ → ^4^I_15/2_, (552 nm) ^4^S_3/2_ → ^4^I_15/2_, (665 nm)^4^F_9/2_ → ^4^I_15/2_, (805 nm) ^4^I_9/2_ → ^4^I_15/2_ transitions in the *f*‐shell of Er^3+^ ions together with nonradiative multiphonon relaxation (Figure 15).

### 2.6 Modified Crystal Field Theory as an Instrument to Describe Energy Levels of the Er^3+^ ions

The energy levels of the free Er^3+^ were calculated using the Modified Crystal Field ‐Theory (MCFT). In this case, a comprehensive orthonormal set of many‐electron symmetry‐adapted functions for the 4*f* ^11^ electron configuration of the free Er^3+[^ ^34^ ^]^ was used. The calculation process involves a careful fitting of the predicted energy levels of the ion to the experimentally measured spectrum. This fitting procedure is accomplished by adjusting two important parameters: the effective nuclear charge ($Z_{eff}^{FI}$) and the *K*_*rel*_ parameter, particularly significant for the interplay of spin‐orbit coupling, as well as other relativistic effects, collectively referred to as Breit's terms.^[^ ^39^ ^]^ By precisely tuning these parameters, we achieve a better alignment between theoretical predictions and experimental observations. This not only improves our insights into the electronic structure of the Er^3+^ ion, but also allows us to make more accurate interpretations of the measured optical characteristics.

A set of parameters for the free ion is a good starting point for calculating the energy levels of the Er^3+^ ion in the crystal field. In this case the $Z_{eff}^{CF}\approx Z_{eff}^{FI}$, since the 4*f* electrons are weakly influenced by a surrounding ligand field due to the strong shielding of the 5*s* ^2^5*p* ^6^ orbitals. However, for the same reason, the ligand charges ($q_{eff}^{CF}$) need to be treated as unknown variables, reduced from their formal oxidation state, $\left(q_{eff}^{CF}=q-\sigma^{CF}\right)$, and could be determined within the MCFT approach.

### 2.7 Energy Levels Calculations of a Free Er^3+^ Ion

It has been found that the best agreement between the calculated and experimental energy levels of the free Er^3+^ ion is achieved at *Z*_*eff*_ = 17.191 and *K*_*rel*_ = 24.728 (Table S14). The nomenclature of the terms and the values of the full moments are given in the first and second columns. The experimental (*E*_*exp*_) and calculated (*E*_*calc*_) values of the multiplets are given in columns three and four. The difference between calculated terms involved in the luminescence process and experimental ones is less than 3% and increases for the higher Er^3+^ levels.^[^ ^41^ ^]^ For only two parameters used in the calculations, it is a very good agreement.

Nevertheless, the calculated free Er^3+^ ion spectrum can only partially describe the observed transitions. For a more accurate identification, it is necessary to consider the crystal environment of the Er^3+^ activator. Indeed, replacing In^3+^ with Er^3+^ should significantly affect the chlorine coordination cage for two reasons. First, the ionic radius of Er^3+^ is about 11% larger than that of In^3+^. Second, the ground state of Er^3+^ is strongly anisotropic ([Xe]4*f* ^11^), while the ground state of In^3+^ ([Kr]4*d* ^10^) has spherical symmetry. While the Er^3+^ concentration is too low for any displacements of Cl ions noticeable in the XRD data, we should add some appropriate distortions of the *O_h_* symmetry to adjust the experimental and calculated optical transitions.^[^ ^34^ ^]^

### 2.8 MCFT Calculations of Luminescence Transitions in the Er^3+^ Ions in the DP Hosts

For the MCFT calculations, the values of the excitation potential (*^V*), the electron‐electron interaction between the valence electrons of Er^3+^ (*^V*_*ee*_), the interaction of the valence electrons with the surrounding crystal field of the chlorine ions (*^V*_*CF*_), and the relativistic interaction between the valence electrons (*^V*_*rel*_) were used. As a basis for the 4*f* ^11^ electronic configuration, the wave functions {Ψ(*SLJM_J_*)} were used, which represent a linear combination of functions Φ (1, 2, …, *n*') composed of the hydrogen‐like functions ψ*_i_* (*n_l_m_l_m_s_*) with the effective parameter *a* = *Z*_eff_/*na*_0_, where *a*_0_ − Bohr radius. The matrix elements of the excitation potential have been calculated numerically, while the order of the secular equation corresponds to the dimension of the many‐determinant functions: $V_{\mu v}=\left\langle \Psi \left(SLJM_{J}\right)\left|\hat{V}\right|\Psi \left(S^{\prime }L^{\prime }J^{\prime }{M^{\prime }}_{J}\right)\right\rangle \left(\mu ,v=1,\text{…},C_{\left(2l+1)(2s+1\right)}^{n^{\prime }}\right)$.

**Figure 16:**
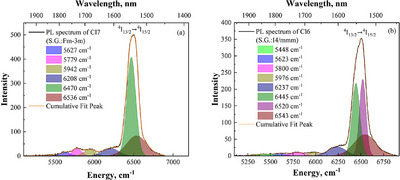
Decomposition of the down conversion PL spectra of the Cs_2_Ag_0.4_Na_0.6_In_0.9_Er_0.01_Sb_0.09_Cl_6_ (Cl7) a) and Cs_2_Ag_0.4_Na_0.6_In_0.9_Er_0.1_Cl_6_ (Cl6) b) samples.

To further investigate the effect of the crystal field on the optical spectrum of Er^3+^, the down‐conversion spectra of the Cs_2_Ag_0.4_Na_0.6_In_0.9_Er_0.01_Sb_0.09_Cl_6_ (Cl7) and Cs_2_Ag_0.4_Na_0.6_In_0.9_Er_0.1_Cl_6_ (Cl6) samples (see Figure 14) were decomposed (Figure 16). The Cl7 sample has a low Er^3+^ concentration, which does not change its cubic structure (S.G. $Fm\bar{3}m$) (Table S1, Figure 16a). The Cl6 sample is 10 times more doped with Er^3+^, which induces the structural transition from cubic to tetragonal symmetry (S.G. I4/mmm) (Table S1, Figure 16b). It is seen that due to the lower symmetry, the number of PL bands in the Cl6 sample is more than that of Cl7. This indicates that the distortions of the Er‐containing coordination complexes are slightly different in the tested samples.

The MCFT calculations show that the multiplets of the ground ^4^I term are almost independent of *q*_*eff*_, *Z*_*eff*_, and *K*_*rel*_ due to the relatively weak crystal field generated by Cl ions. The splitting of the Er^3+^ levels by the cubic and tetragonal crystal fields (black solid lines) does not exceed 150 and 650 cm^−1^, respectively, at *q*_*eff*_ = ‐1. Therefore, we can use the following parameters to calculate the energy levels of the ^4^I term: *q*_*eff*_ =  ‐1, *Z*_*eff*_  =  17.191, and *K*_*rel*_ = 24.728.

**Figure 17:**
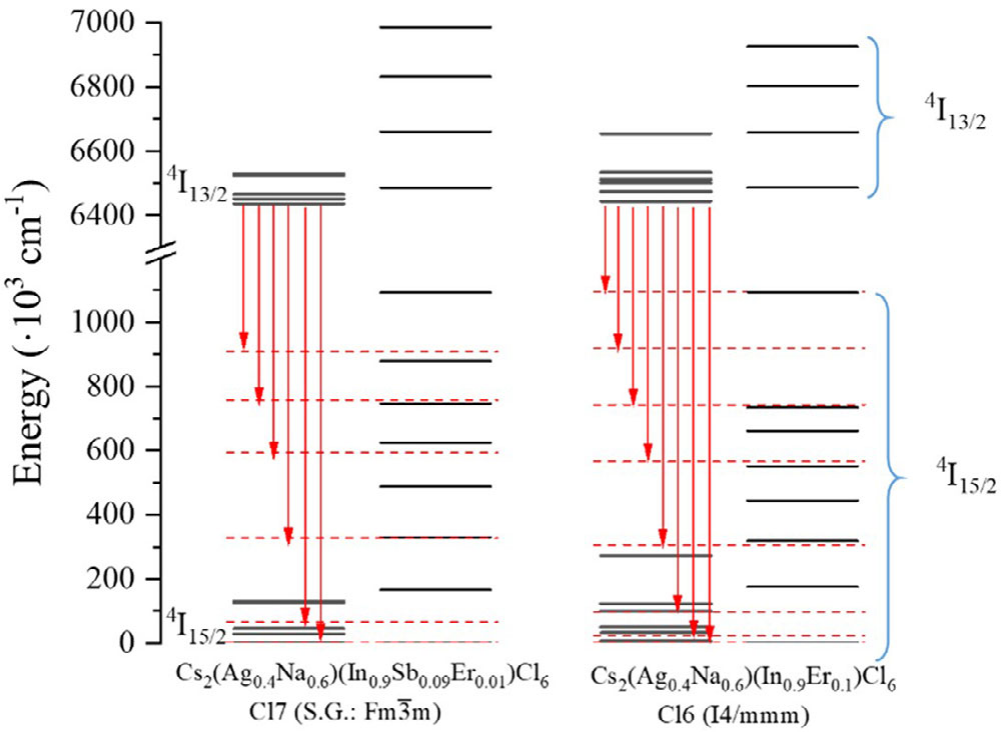
Energy levels of Er^3+^ ions embedded in the chloride DP matrices (Cl6 and Cl7). The first and the third columns: the calculation has been performed for [ErCl_6_]^3‐^ in the cubic and tetragonal symmetry crystals; the second and fourth columns: the calculation has been performed for [ErCl_6_]^3‐^ subjected to additional distortions.

The crystal field energy levels of Er^3+^ ions embedded in the chloride DP matrices (Cl6 and Cl7) were calculated, considering that Er^3+^ replaces In^3+^ and forms the six‐coordinated [ErCl_6_]^3‐^ complexes (Figure 17). According to the MCFT approach, the ligands in the [ErCl_6_]^3‐^ coordination complex are fixed point charges with the chlorine oxidation state of ‐1, the coordinates of the ligands are taken from the XRD data (see Table S4–S7). Columns 1 and 3 in Figure 17 represent the energy levels of Er^3+^ calculated in the cubic and tetragonal symmetry crystals. Columns 2 and 4 are calculated for the [ErCl_6_]^3‐^ coordination complexes subjected to additional distortions. Due to the cubic symmetry, the [ErCl_6_]^3‐^ coordination complexes are regular, and the charges of the ligands can be assumed to be equal. In the case of the tetragonal symmetry, *q*_*eff*_ are equal in pairs.

The experimental ^4^I_13/2_ → ^4^I_15/2_ transitions marked by the red solid arrows are observed in the range of (0−906) and (0−1089) cm^−1^ in the Cs_2_Ag_0.4_Na_0.6_In_0.9_Er_0.01_Sb_0.09_Cl_6_ (Cl7) and Cs_2_Ag_0.4_Na_0.6_In_0.9_Er_0.1_Cl_6_ (Cl6) samples (see Figure 16 and columns 1 and 3 in Figure 17). Thus, to simulate the experimentally observed values, we added some *O_h_* appropriate distortions to the initial structures. The MCFT calculations of the PL transitions show that, in the case of the Cl6 sample, the better agreement can be obtained when the [ErCl_6_]^3‐^ octahedra are subjected to the Jahn‐Teller distortions of both types (*Q*_2_ and *Q*_3_). The classification of the *O_h_* point group distortions is the same as in the literature^[^ ^42^ ^]^ and to the full symmetry breathing, like *Q*_1_ distortions. In the case of the Cs_2_Ag_0.4_Na_0.6_In_0.9_Er_0.01_Sb_0.09_Cl_6_ (Cl7) sample, the Jahn‐Teller distortions of only *Q*_2_‐type are necessary, and the *Q*_1_ breathing‐type distortions should also be applied. Note that the agreement is quite good, considering that the widths of the bands are in the range of 60–250 cm^−1^ and 70–240 cm^−1^, respectively.

**Figure 18:**
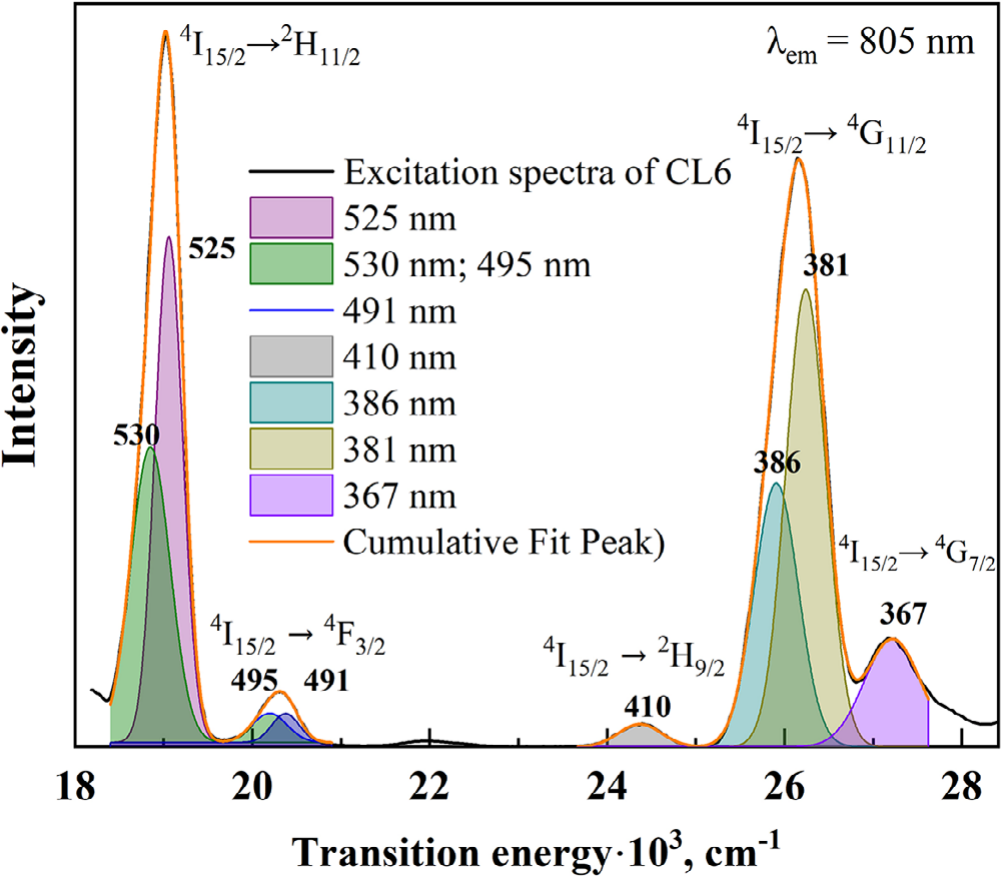
Decomposition of the excitation spectrum of the Cs_2_Ag_0.4_Na_0.6_In_0.9_Er_0.1_Cl_6_ (Cl6) sample.

**Figure 19:**
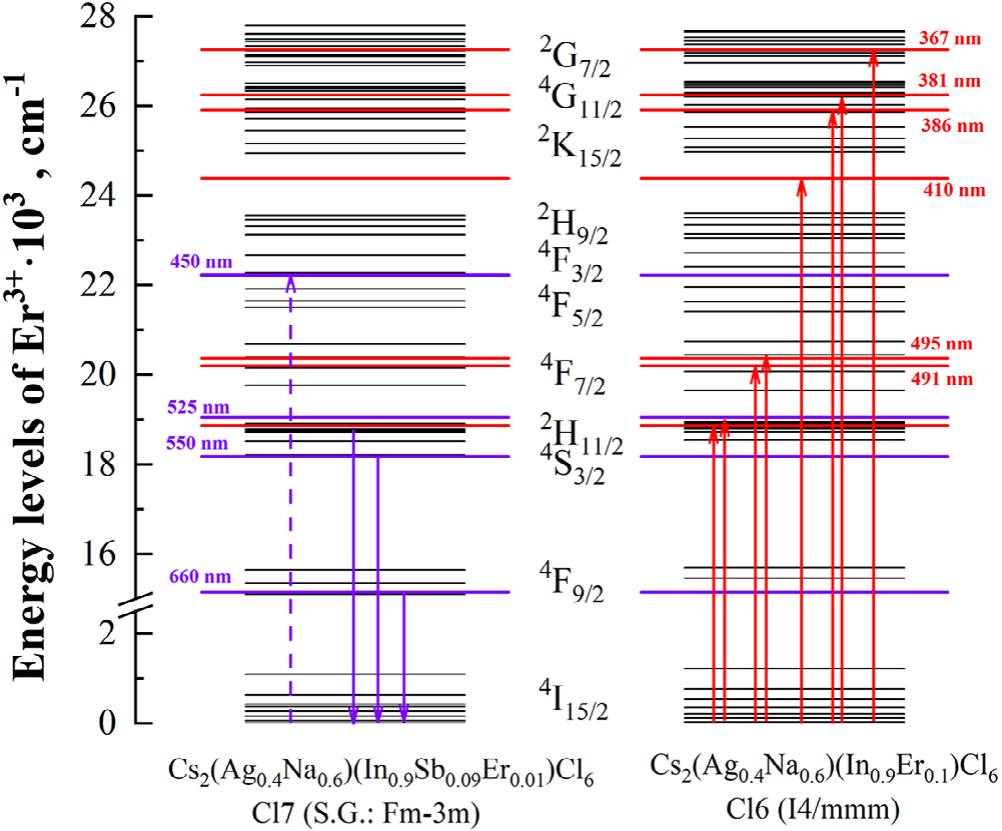
Calculated energy levels of [ErCl_6_]^3‐^ in the cubic Cs_2_Ag_0.4_Na_0.6_In_0.9_Er_0.01_Sb_0.09_Cl_6_ (Cl7) and tetragonal Cs_2_Ag_0.4_Na_0.6_In_0.9_Er_0.1_Cl_6_ (Cl6) samples.

Therefore, the doping‐induced deformation of the Er^3+^‐containing coordination complex leads to the concentration quenching observed in the tested samples (Figure 14). The changes in the intensity of the optical bands are caused by the rearrangement of the Er energy levels and, consequently, lead to the rise of the nonradioactive transitions. To clearly visualize the energy levels of the excited bands (see Figure 12), we have decomposed the Cl6 excitation spectrum and calculated the Er^3+^ energy levels for the Cl6 and Cl7 samples. The decomposed spectrum is shown in Figure 18, and the calculated Er^3+^ energy levels are shown up to 28·10^3^ cm^−1^ (Figure 19).

Considering that the excited terms depend slightly on *Z*_*eff*_ and *K*_*rel*_,^[^ ^42^ ^]^ we fit the following parameters to calculate the Er^3+^ excited terms in the Cl6 and Cl7 samples: *q*_*eff*_ =  ‐1, *Z*_*eff*_  =  15.9, and *K*_*rel*_ = 24.1 and the same as before distortions of the [ErCl_6_]^3‐^ coordination complexes. The violet dashed arrow in Figure 19 denotes the absorption transition caused by the Sb^3+^ emission at 450 nm. The violet solid arrows are the emission transitions in Er^3+^, as seen in Figure 13. The red solid lines are the excitation transitions in Er^3+^ and they are shown in Figure 12.

The MCFT calculations indicate that distortions of the Er^3+^‐containing complexes have a strong effect on the intensities of the observed transitions. As shown in Figure 13, the emission transition induced by Sb^3+^ is the most intense in Cs_2_Ag_0.4_Na_0.6_In_0.9_Er_0.01_Sb_0.09_Cl_6_ (Cl7). The nearly absent excitation band at 410 nm in Cl6 can be attributed to the rearrangement of the crystal structure to tetragonal in Cs_2_Ag_0.4_Na_0.6_In_0.9_Er_0.1_Cl_6_ (Cl6) caused by the most Er doping, which alters the energy levels of Er^3^⁺. While for the structure of Cs_2_Ag_0.4_Na_0.6_In_0.9_Er_0.07_Sb_0.03_Cl_6_ (Cl9), the excitation band at 410 nm is the highest, the Er^3+^ concentration is a little lower than in Cl6. It means that symmetry plays a significant role in the emission spectra of Er^3+^ in chloride DPs. Figure 19 illustrates that 410 nm band lies between the ^2^H_9/2_ and ^2^K_15/2_ levels, and is not excited with 805 nm in Cl6 sample (see Figure 12).

### 2.9 LED Production

**Figure 20:**
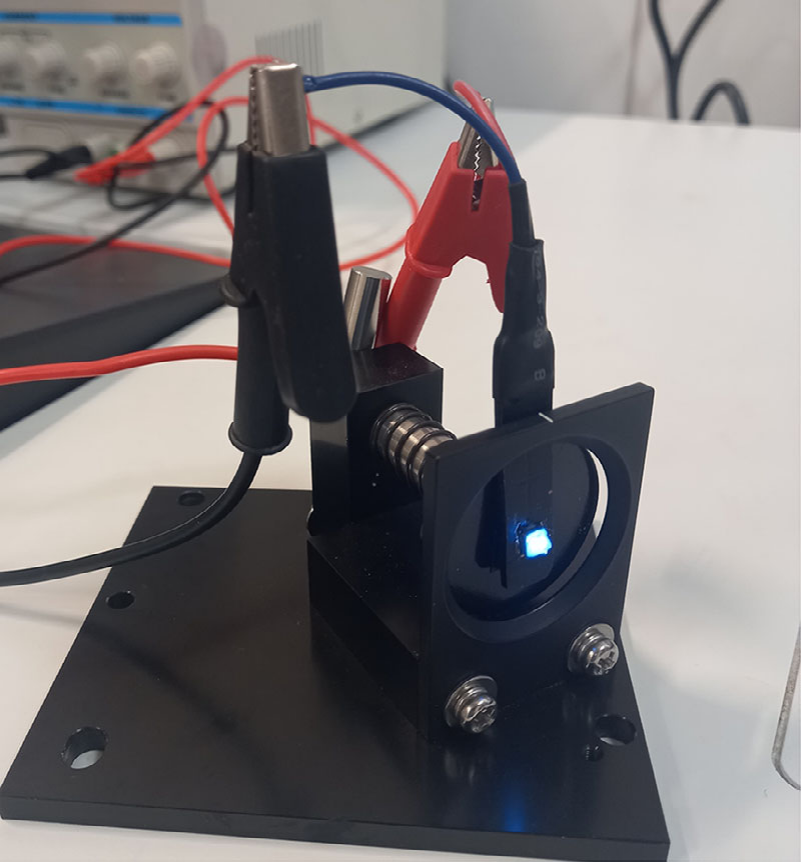
Photograph of the LED lamp fabricated with a UV chip (320 nm) under bias (4.4 V, 0.02 A) during the measurement.

**Figure 21:**
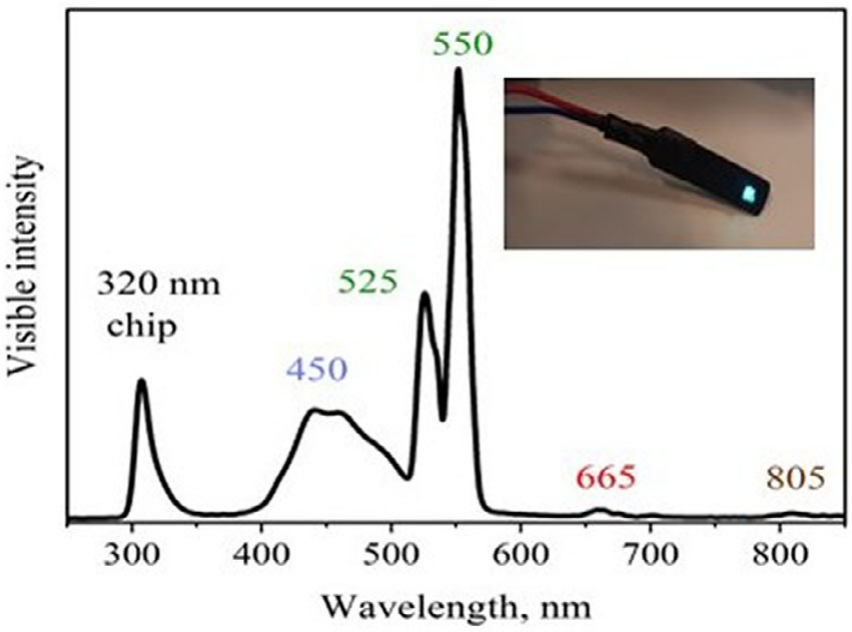
Measured EL spectrum of the LED lamp fabricated using the UV‐chip (320 nm) and synthesized Cs_2_Ag_0.4_Na_0.6_In_0.9_Er_0.01_Sb_0.09_Cl_6_ (Cl7) powder sample as the luminophore material and photo of it under bias (4.4 V, 0.02 A).

**Figure 22:**
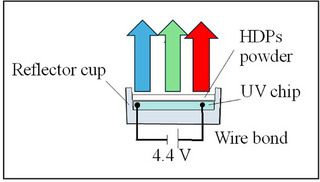
Schematic structure of the LED.

To fabricate an LED lamp, Cs_2_Ag_0.4_Na_0.6_In_0.9_Er_0.01_Sb_0.09_Cl_6_ (Cl7) sample as luminophore material was mixed with epoxy resin at room temperature. Then, the mixture was put on the commercial UV LED at wavelength of emission 320 nm (Figures 20, 21, 22).

Under 4.4 V bias voltage, the prepared LED emits blue and green light. The bottom‐left image in Figure 21 shows the emission of the LED in the dark. The spectral profile of the LED in the Vis‐ and NIR region is like the measured PL spectrum of the Cl7 sample (see Figure 13). The obtained results are in good agreement with data by Gray et al.,^[^ ^38^ ^]^ who investigated that the stability of the phosphor‐converted light‐emitting diode (pc‐LED) based on Cs_2_NaInCl_6_ doped with Er^3+^/Sb^3+^ is high with stable blue and short‐wave infrared radiation (SWIR).

## 3 Conclusion

We have synthesized stable undoped chloride DPs (Cs_2_AgInCl_6_, Cs_2_Ag_0.4_Na_0.6_InCl_6_, Cs_2_NaInCl_6_), Er^3+^‐doped, and Sb^3+^–Er^3+^co‐doped Cs_2_Ag_0.4_Na_0.6_InCl_6_ using solid‐state techniques. The dopants were found to replace the In^3+^ ions in the host lattice of the chloride DPs. Samples containing Sb^3+^ show optical absorptions at sub‐band gap energies due to 5*s* ^2^→5*s* ^1^5*p* ^1^ electronic transitions classified as A‐, B‐, and C‐bands, then the Sb^3+^ states de‐excite by emitting blue light (450 nm). Co‐doping of Sb^3+^ and Er^3+^ leads to the transfer of excitation energy from Sb^3+^ to Er^3+^. Then, the excited *f*‐electrons in Er^3+^ de‐excite by emitting sharp spectral lines in both the Vis‐ and NIR regions. Therefore, under 320 nm radiation, the PL spectra can be obtained from the emission of Er^3+^ and Sb‐doped perovskites. Excitation at the resonance wavelength (805 nm for Er^3+^) results in the appearance of PL bands (1540 nm) associated with erbium ions. A significant increase in the erbium content can lead to concentration quenching of PL due to an increase in the probability of nonradiative transitions. The MCFT calculations indicate that distortions of the Er^3+^‐containing complexes strongly affect the Er transition intensities. Rearrangement of the crystal structure from cubic $Fm\bar{3}m$ to the tetragonal I4/mmm phase can be caused by the most Er doping, which alters the energy levels of Er^3+^. This means that symmetry plays a significant role in the emission spectra of Er^3+^ in chloride DPs. A prototype of the LED, based on the UV chip (320 nm) and synthesized Cs_2_Ag_0.4_Na_0.6_In_0.9_Er_0.01_Sb_0.09_Cl_6_ powder sample as the luminophore material, has been prepared. The emission spectrum has been measured under 4.4 V bias voltage, and the prepared LED emits blue and green light because of Sb^3+^ and Er^3+^ emission. We can conclude that it is possible to obtain the efficient stable multimodal emission in Sb^3+^−Er^3+^‐co‐doped Cs_2_Ag_0.4_Na_0.6_InCl_6_ chloride DPs using down‐conversion to cover the Vis‐near‐IR spectral range.

## Author Contribution

All authors of the manuscript have checked the manuscript and have agreed to the submission

Deposition Numbers CCDC‐2450850 (for Cs_2_Ag_0.4_Na_0.6_InCl_6_), CCDC‐2450876 (for Cs_2_Ag_0.4_Na_0.6_In_0.9_Er_0.1_Cl_6_), CCDC‐2450891 (for Cs_2_Ag_0.4_Na_0.6_In_0.9_Sb_0.09_Er_0.01_Cl_6_) contain the supplementary crystallographic data for this paper. These data are provided free of charge by the joint Cambridge Crystallographic Data Centre via www.ccdc.cam.ac.uk/data_request/cif. and Fachinformationszentrum Karlsruhe (http://www.fiz‐karlsruhe.de/).

## Conflict of Interests

The authors declare no conflict of interest.

## Supporting information



Supporting Information

Supporting Information

## Data Availability

Data associated with this paper are downloadable from https://doi.org/10.58099/PK/RYPCF7.
