# Supplementary material for: Chloride Double Perovskites Doped With Sb3+/Er3+ as Stable and Effective Luminescence Material in the Vis‐NIR Region
Source: Chemistry. 2025 Jun 8;31(36):e202500066. doi: 10.1002/chem.202500066 (PMC12202846; doi:10.1002/chem.202500066)
Supplement: Supplementary file 1 — Supporting Information [file CHEM-31-e202500066-s001.docx]

**Chloride Double Perovskites Doped with Sb^3+^/Er^3+^ as Stable and Effective Luminescence Material in the Vis-NIR Region**

I. A. Ivashchenko^1^, V. V. Halyan^2^, L. D. Gulay^2^, V.O. Yukchymchuk^3^, A. Eich^4^, M. Rusu^4^, G. Gurieva^4^, S. Schorr^4,11^, P. Dąbczyński^5^, Yu. Kazarinov^6^, K.V. Lamonova^7,8^, O. Khyzhun^9,10^ and K. Matras-Postołek^*1^

*^1^Cracow University of Technology, Faculty of Chemical Engineering and Technology, Warszawska St. 24, 31-155 Cracow, Poland*

*^2^Lesya Ukrainka Volyn National University, Voli Ave. 13, 43000 Lutsk, Ukraine*

*^3^V.E. Lahskaryov Institute of Semiconductor Physics, National Academy of Sciences, Nauky ave 41, 03028 Kyiv, Ukraine*

*^4^Helmholtz-Zentrum Berlin für Materialien und Energie, Berlin, Germany*

*^11^Freie Universität Berlin, Institut of Geological Sciences, Berlin, Germany*

*^5^Jagiellonian University, Faculty of Physics, Astronomy and Applied Computer Science, Łojasiewicza, 11, 30-348 Cracow, Poland*

*^6^NSC Kharkiv Institute of Physics and Technology, Akademichna str. 1, 61108 Kharkiv, Ukraine*

*^7^Max-Born-Institute for Nonlinear Optics and Short Pulse Spectroscopy, Max-Born-Straße, 2A, 12489 Berlin, Germany*

*^8^O. O. Galkin Donetsk Institute for Physics and Engineering, NAS of Ukraine, Nauky ave. 46, 03028 Kyiv, Ukraine*

*^9^ Frantsevich Institute for Materials Science, National Academy of Sciences, Omeliana Pritsaka (Krzhizhanovsky) str., 3, 03142 Kyiv, Ukraine*

*^10^Jan Długosz University, Armii Krajowej 13/15,42200 Czestochowa, Poland*

^*^*Email: prof. K. Matras-Postołek (k.matras@pk.edu.pl*

**Content:**

**1. Synthesis and methods of investigation**

**Table S1.** Compositions and structure data of the synthesized samples obtained with different method of investigation.

**Fig. S1** Experimental (dots), calculated (solid) and difference (bottom scale) diffractograms of the chloride DP powder samples.

**Table S2.** Results of crystal structure refinement of the chloride DP powder samples.

**Тable S3.** Refined coordinates of atoms and their isotropic thermal parameters in the chloride DP powder samples.

**Table S4**. Interatomic distances and coordination numbers (C.N.) of atoms in the structures of the chloride DP powder samples.

**Fig. S2** Powder X-ray diffraction patterns of the doped chloride DPs: experimental (dots), calculated (solid) and difference (bottom scale) diffractograms.

**Table S5.** Results of the crystal structure refinement of the chloride DP doped powder samples.

**Тable S6.** Refined coordinates of atoms and their isotropic thermal parameters in the chloride DP doped powder samples.

**Table S7.** Interatomic distances and coordination numbers (C.N.) of atoms in the chloride DP doped powder samples.

**Table S8.** Batch and analytical compositions of the obtained powder samples.

**Table S9.** The crystallographic characteristics and the details of the refinement of the structures of the Cs_2_Ag_0.4_Na_0.6_InCl_6_,

Cs_2_Ag_0.4_Na_0.6_In_0.9_Cl_6_ 0.1ErCl_3_ (6Cl) and Cs_2_Ag_0.4_Na_0.6_In_0.9_Cl_6_ 0.09SbCl_3_ 0.01ErCl_3_ (7Cl) samples (single crystal XRD method).

**Table S10.** Atomic coordinates in the structure of the single crystals Cs_2_Ag_0.292_Na_0.708_InCl_6_, Cs_2_Ag_0.285_Na_0.715_In_0.971_Er_0.029_Cl_6_, Cs_2_Ag_0.160_Na_0.840_In_0.893_Er_0.017_Sb_0.090_Cl_6_ (single crystal XRD method).

**Table S11.** Anisotropic parameters of the atoms in the structure of the single crystals Cs_2_Ag_0.292_Na_0.708_InCl_6_, Cs_2_Ag_0.285_Na_0.715_In_0.971_Er_0.029_Cl_6_, Cs_2_Ag_0.160_Na_0.840_In_0.893_Er_0.017_Sb_0.090_Cl_6_ (single crystal XRD method).

**Table S12.** Interatomic distances (d, Å) and coordination numbers (C.N.) of the atoms in the structure of the single crystals Cs_2_Ag_0.292_Na_0.708_InCl_6_, Cs_2_Ag_0.285_Na_0.715_In_0.971_Er_0.029_Cl_6_, Cs_2_Ag_0.160_Na_0.840_In_0.893_Er_0.017_Sb_0.090_Cl_6_ (single crystal XRD method).

**Table S13.** Batch and analytical compositions of the single crystals.

**Fig. S3** SEM photos of the Cl6, Cl7, Cl8 samples and their EDS results.

**Fig. S4** Maps of the elements of the Cs_2_Ag_0.4_Na_0.6_In_0.9_Sb_0.09_Er_0.01_Cl_6_ (Cl7) powder sample.

**Fig. S5** Maps of the elements of the Cl6 single crystal Cs_2_Ag_0.285_Na_0.715_In_0.971_Er_0.029_Cl_6_ (Cs_2_Ag_0.4_Na_0.6_In_0.9_Er_0.1_Cl_6_ batch composition).

**Fig. S6.** ToF-SIMS mass spectra collected for Cs_2_Ag_0.4_Na_0.6_InCl_6_ (black line), Cs_2_Ag_0.4_Na_0.6_In_0.9_Sb_0.09_Er_0.01_Cl_6_ (Cl7) (blue line), Cs_2_Ag_0.4_Na_0.6_In_0.9_Sb_0.05_Er_0.05_Cl_6_ (Cl8) (orange line), Cs_2_Ag_0.4_Na_0.6_In_0.9_Sb_0.1_Cl_6_ (magenta line), Cs_2_Ag_0.4_Na_0.6_In_0.9_Er_0.1_Cl_6_ (Cl6) (green line). The area of the elements of interest are highlighted.

**Fig. S7.** Mass spectra from the area of Er^+^ signal plotted for all samples.

**Fig. S8.** Mass spectra from the area of Sb^+^ signal plotted for all samples.

**Fig. S9.** Survey XPS spectra of (1) Cs_2_Ag_0.4_Na_0.6_InCl_6_, (2) Cs_2_Ag_0.4_Na_0.6_In_0.9_Sb_0.1_Cl_6_, (3) Cs_2_Ag_0.4_Na_0.6_In_0.9_Sb_0.09_Er_0.01_Cl_6_, and (4) Cs_2_Ag_0.4_Na_0.6_In_0.9_Sb_0.05_Er_0.05_Cl_6_ (Note: due to several XPS spectral features, only the most important ones are marked).

**Fig. S10.** XPS core-level spectra of (1) Cs_2_Ag_0.4_Na_0.6_InCl_6_, (2) Cs_2_Ag_0.4_Na_0.6_In_0.9_Sb_0.1_Cl_6_, (3) Cs_2_Ag_0.4_Na_0.6_In_0.9_Sb_0.09_Er_0.01_Cl_6_, and (4) Cs_2_Ag_0.4_Na_0.6_In_0.9_Sb_0.05_Er_0.05_Cl_6_: (a, b) Cs 3*d*, (c, d) Cl 2*p*, (e, f) Ag 3*d*, and (g, h) In 3*d* (Note: due to a number of XPS spectral features, only the most important ones are marked).

**Table S14.** Experimental (*E*_exp_) [38] and calculated^*^ (*E*_calc_) energy levels of a free Er^3+^ ion.

**1. Synthesis and methods of investigation**

The chloride double perovskites (DPs) Cs_2_AgInCl_6_, Cs_2_NaInCl_6_, Cs_2_Ag_0.4_Na_0.6_InCl_6_, and Cs_2_Ag_0.4_Na_0.6_In_0.9_Cl_6_:0.1-*x*Er^3+^; *x*Sb^3+^, *x* = 0; 0.03; 0.05; 0.09 were synthesized by the solid-state technique. The starting chemicals (CsCl (99.5 wt.%), AgCl (99.99 wt.%), NaCl (99.9 wt.%), InCl_3_ (99.99 wt.%), SbCl_3_ (99.95 wt.%) and ErCl_3_ (99.9 wt.%) were commercially purchased from Sigma Aldrich (USA). In a typical Cs_2_Ag_0.4_Na_0.6_In_0.9_Cl_6_:0.1Er^3+^ sample, 0.5144 g of CsCl, 0.0873 g of AgCl, 0.0534 g of NaCl, 0.3032 g of InCl_3_ and 0.0417 g of ErCl_3_ were weighed using analytical lab balance with accuracy till 4 sighs after comma. Then the mixtures were transferred into quartz tubes which were sealed under the residual pressure 0.133 Pa. The synthesis was performed in a shaft-type furnace with temperature control with an accuracy of ± 5K. The maximum synthesis temperature was 870 K (the heating rate was 10 K/h), annealing for 24 hours and quick cooling with the furnace off. After the first stage of the synthesis, it was necessary to repeat the procedure after grinding the alloys into powder. After a heating to 870 K and annealing the samples for 10 hours, they were quenched into 25 % aqueous NaCl solution. The obtained alloys have been investigated by X-ray powder diffraction (XRD) (using the SmartLab powder diffractometer, CuKα radiation, 2θ-range of 10-120°, 0.05° scan step). The crystal structure of the HDPs were evaluated applying the powder method using the WinCSD software [RS1]. For the registration of the Raman signals, an MDR-23 spectrometer equipped with a cooled CCD detector iDus 420 Andor (UK) was used. The Raman signals were excited by the diode-pumped solid-state laser lines (λ_exc_) at 457, 532, and 671 nm. The excitation of Raman spectra by radiation with different wavelengths enables the realisation of resonant Raman scattering or at least approaches it, leading to a significant increase in the intensities of specific bands. This is especially important if the materials have a complex elemental composition, as is the case in our situation. As secondary phases can form in these materials, certain types of radiation may be resonant and allow for their detection. The laser power density on the sample surface was less than 10^3^ W/cm^2^, to preclude any thermal modification of the samples. The spectral resolution for all excitation radiation wavelengths did not exceed 4 cm^-1^ and was determined from the Si phonon peak width of a Si single crystal. The Si phonon peak position of 520.5 cm^-1^ was used as a reference for determining the position of the peaks in the Raman spectra acquired at different λ_exc_.

The chloride DPs crystal shape and sizes were studied using an Apreo 2s LoVac (Thermo Fisher Scientific) scanning electron microscope. The element distribution was determined by means of energy-dispersive X-ray spectroscopy.

Time-of-flight Secondary Ion Mass Spectrometry (ToF–SIMS) experiments were performed on an IONTOF ToF-SIMS V (Munster, Germany) instrument, equipped with a 30 keV bismuth–manganese liquid metal ion source. Samples were analyzed with a focused Bi_1_^+^ ion beam scanned over 150×150 μm^2^ at least for three spots per sample. The primary ion current was fixed at 1.3 pA, which gives for each experiment total ion dose density of 1×10^12^ cm^-2^. Mass calibration for each measurement was performed with CH^+^, C_2_H_2_^+^, C_3_H_3_^+^, C_4_H_4_^+^, C_5_H_5_^+^ positive mass spectra. The peaks were labelled to a deviation of mass shift between the theoretical labelled mass and measured mass no larger than ±100 ppm. Powder samples were spread on the conductive carbon tape.

The absorption spectra were recorded on a Shimadzu UV-2600 UV-Vis spectrophotometer. A Hitachi F-4600 fluorescence spectrometer was used to measure the emission and excitation spectra of the samples. The PL spectra in the near-IR region were measured using an MDR-204 monochromator. The output signal was recorded by Si and PbS photosensors. The emission of laser with the wavelength at 805 nm were used for the excitation of PL.

The specimens were tested also employing possibilities of X-ray photoelectron spectroscopy (XPS). Measurements of the XPS spectra of the samples under study were made using a Perkin Elmer spectrometer equipped with a monochromatized Al Kα source (*E*=1486.6 eV). Since the XPS method is known to be very sensitive to surface charging effects, they were compensated in the present work by using a special flood gun equipped with the spectrometer.

**Table S1.** Compositions and structure data of the synthesized samples obtained with different method of investigation

| Composition of the sample | Number | Crystal structure, lattice parameters, Å | |
| --- | --- | --- | --- |
|  |  | Powder XRD | Single crystal XRD |
| Cs_2_AgInCl_6_ | 1 | $Fm\bar{3}m$*, a* = 10.4806(2) Å | --- |
| Cs_2_Ag_0.4_Na_0.6_InCl_6_ | 2 | $Fm\bar{3}m$*, a* = 10.5114(2) Å | $Fm\bar{3}m$*, a*= 10.5008(3) Å |
| Cs_2_NaInCl_6_ | 3 | $Fm\bar{3}m$*, a* = 10.5359(2) Å | --- |
| Cs_2_Ag_0.4_Na_0.6_In_0.9_Er_0.1_Cl_6_ | Cl6 | *I4/mmm, a* = 7.4449(1), *c* = 10.5163(3) Å | $Fm\bar{3}m$*, a*=10.5018(2) Å |
| Cs_2_Ag_0.4_Na_0.6_In_0.9_Sb_0.09_Er_0.01_Cl_6_ | Cl7 | $Fm\bar{3}m$*, a* = 10.5271(1) Å | $Fm\bar{3}m$*, a*=10.5148(2) Å |
| Cs_2_Ag_0.4_Na_0.6_In_0.9_Sb_0.05_Er_0.05_Cl_6_ | Cl8 | *I4/mmm, a* = 7.4494(1), *c* = 10.5193(3) Å | --- |
| Cs_2_Ag_0.4_Na_0.6_In_0.9_Sb_0.03_Er_0.07_Cl_6_ | Cl9 | only phase analysis performed | --- |


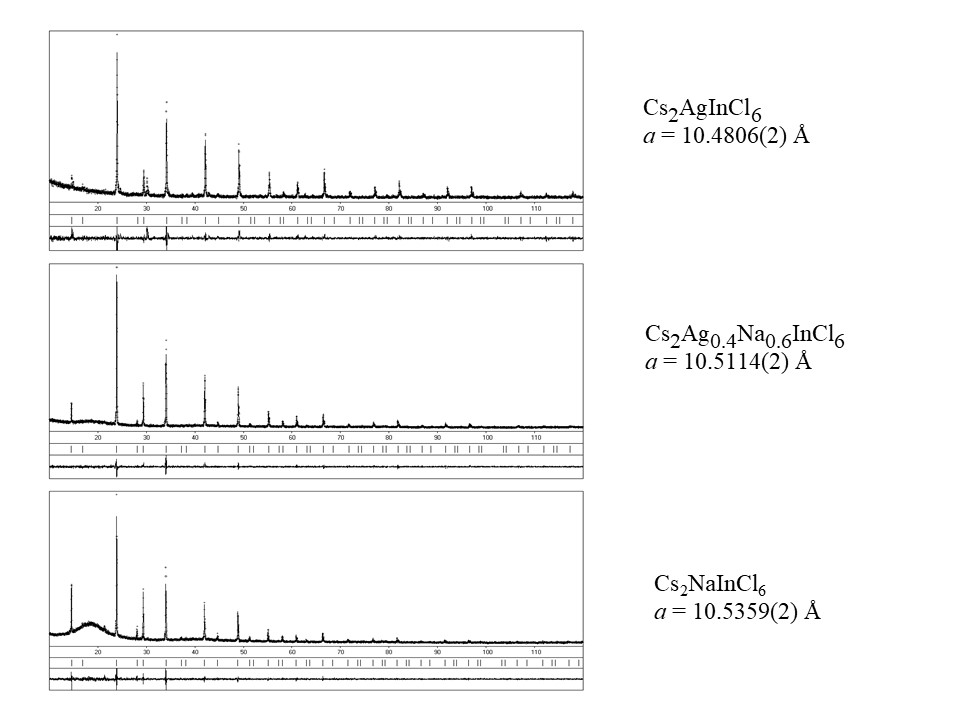


**Fig. S1** Experimental (dots), calculated (solid) and difference (bottom scale) diffractograms of the chloride DP powder samples.

**Table S2.** Results of crystal structure refinement of the chloride DP powder samples

| Empirical Formula | Cs_2_AgInCl_6_ | Cs_2_Ag_0.4_Na_0.6_InCl_6_ | Cs_2_NaInCl_6_ |
| --- | --- | --- | --- |
| Sp. Gr; Z | $Fm\bar{3}m$; 4 | $Fm\bar{3}m$; 4 | $Fm\bar{3}m$; 4 |
| Unit cell parameters, *Å* | a = 10.4806(2) | a = 10.5114(2) | a = 10.5359(2) |
| *V*,  *Å* ^3^ | 1151.21(7) | 1161.40(6) | 1169.52(7) |
| Number of atoms in cell | 40 | 40 | 40 |
| Calculated density (g/cm^3^) | 4.0454(2) | 3.7187(2) | 3.5001(2) |
| Absorption coefficient (1/cm) | 914.39 | 828.05 | 770.46 |
| Radiation and wavelenght ( *Å* ) | CuKα; 1.54185 | CuKα; 1.54185 | CuKα; 1.54185 |
| Diffractometer | SmartLab, Rigaku | SmartLab, Rigaku | SmartLab, Rigaku |
| Mode of refinement | Full profile | Full profile | Full profile |
| Number of free parameters | 6 | 6 | 6 |
| *R_І_*; *R_р_* | 0.0655; 0.2737 | 0.0470; 0.2003 | 0.0460; 0.2477 |
| Scale factor | 0.6163(10) | 0.4859(2) | 0.5974(6) |
| Texture axis and parameter | [ 0 1 3 ] -0.48(3) | [ 0 1 3 ] 1.26(2) | [ 0 1 3 ] 0.94(3) |

**Тable S3.** Refined coordinates of atoms and their isotropic thermal parameters in the chloride DP powder samples

| Cs_2_AgInCl_6_ | | | | | | |
| --- | --- | --- | --- | --- | --- | --- |
| Atom | Wyck off positions | *x/a* | *y/b* | *z/c* | Occupation | В_iso._, Å^2^ |
| Cs | 8*c* | 1/4 | 1/4 | 1/4 | 1 | 1.28(14) |
| Ag | 4*b* | 1/2 | 1/2 | 1/2 | 1 | 0.3(3) |
| In | 4*d* | 0 | 0 | 0 | 1 | 0.7(3) |
| Cl | 24*e* | 0.237(2) | 0 | 0 | 1 | 0.2(2) |
| Cs_2_Ag_0.4_Na_0.6_InCl_6_ | | | | | | |
| Atom | Wyck off positions | *x/a* | *y/b* | *z/c* | Occupation | В_iso._, Å^2^ |
| Cs | 8*c* | 1/4 | 1/4 | 1/4 | 1 | 3.5(2) |
| M | 4*b* | 1/2 | 1/2 | 1/2 | 0.4Ag+0.6Na | 3.9(5) |
| In | 4*d* | 0 | 0 | 0 | 1 | 2.9(3) |
| Cl | 24*e* | 0.2364(11) | 0 | 0 | 1 | 3.1(3) |
| Cs_2_NaInCl_6_ | | | | | | |
| Atom | Wyck off positions | *x/a* | *y/b* | *z/c* | Occupation | В_iso._, Å^2^ |
| Cs | 8*c* | 1/4 | 1/4 | 1/4 | 1 | 4.4(2) |
| Na | 4*b* | 1/2 | 1/2 | 1/2 | 1 | 2.9(11) |
| In | 4*d* | 0 | 0 | 0 | 1 | 3.4(3) |
| Cl | 24*e* | 0.2303(11) | 0 | 0 | 1 | 3.6(3) |

**Table S4.** Interatomic distances and coordination numbers (C.N.) of atoms in the structures of the chloride DP powder samples

| Atoms | Cs_2_AgInCl_6_ | Cs_2_Ag_0.4_Na_0.6_InCl_6_ | Cs_2_NaInCl_6_ | C.N. |
| --- | --- | --- | --- | --- |
|  | Interatomic distances, Å | | |  |
| M– 6Cl | 2.76(2) | 2.771(11) | 2.841(12) | 6 |
| In– 6Cl | 2.48(2) | 2.485(11) | 2.427(12) | 6 |
| Cs– 12Cl | 3.7080(7) | 3.7191(5) | 3.7307(7) | 12 |


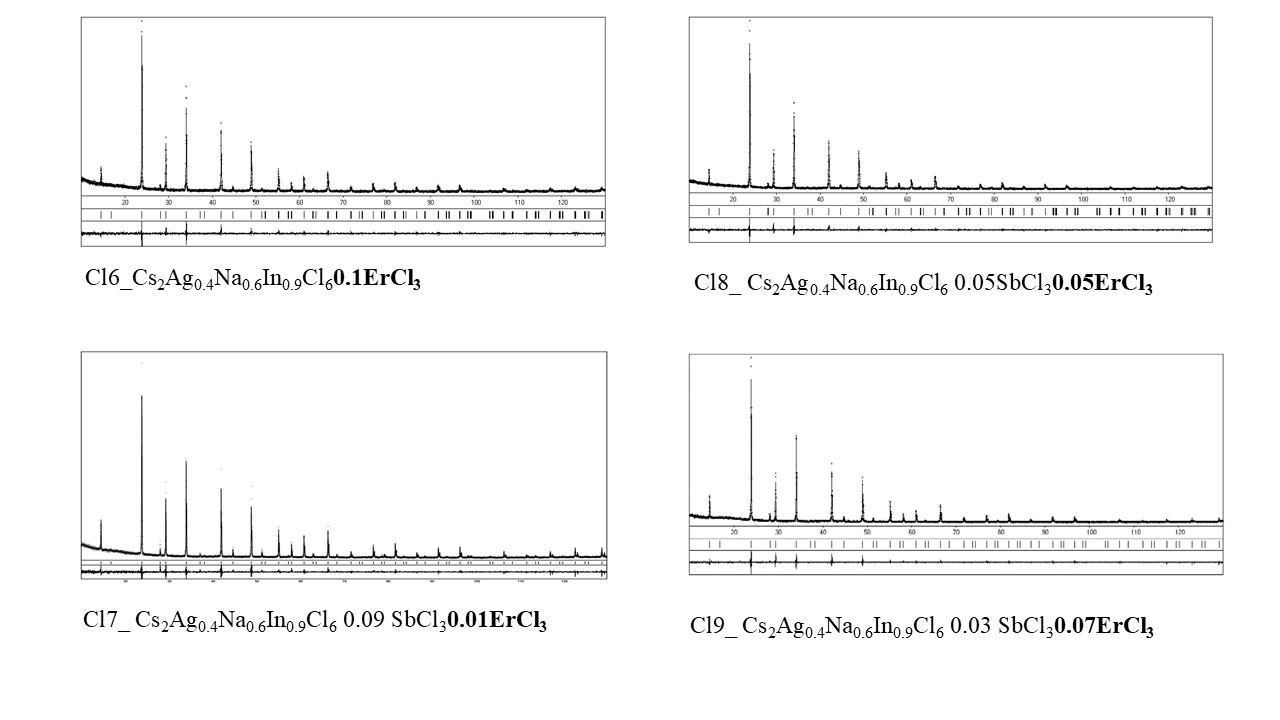


**Fig. S2** Powder X-ray diffraction patterns of the doped chloride DPs: experimental (dots), calculated (solid) and difference (bottom scale) diffractograms.

| Empirical Formula | (Cl6) Cs_2_Ag_0.4_Na_0.6_In_0.9_Er_0.1_Cl_6_ | (Cl7) Cs_2_Ag_0.4_Na_0.6_In_0.9_Sb_0.09_Er_0.01_Cl_6_ | (Cl8) Cs_2_Ag_0.4_Na_0.6_In_0.9_Sb_0.05_Er_0.05_Cl_6_ |
| --- | --- | --- | --- |
| Sp. Gr; Z | *I*4*/mmm*; 2 | $Fm\bar{3}m$; 4 | *I*4*/mmm*; 2 |
| Unit cell parameters, *Å* | a = 7.4449(1)  c = 10.5163(3) | a = 10.5271(1) | a = 7.4494(1)  c = 10.5193(3) |
| *V*,  *Å* ^3^ | 582.88(4) | 1166.62(4) | 583.75(4) |
| Number of atoms in cell | 20 | 40 | 20 |
| Calculated density (g/cm^3^) | 3.7347(3) | 3.7086(2) | 3.7162(2) |
| Absorption coefficient(1/cm) | 821.83 | 826.58 | 823.58 |
| Radiation and wavelenght | CuKα; 1.54185 Å | CuKα;1.54185 Å | CuKα; 1.54185 Å |
| Diffractometer | SmartLab, Rigaku | SmartLab, Rigaku | SmartLab, Rigaku |
| Mode of refinement | Full profile | Full profile | Full profile |
| Number of free parameters | 9 | 7 | 9 |
| *R_І_*; *R_р_* | 0.0607; 0.2333 | 0.0580; 0.2571 | 0.0466; 0.2022 |
| Scale factor | 0.17292 | 0.11179 | 0.14916 |
| Texture axis and parameter | [ 0 1 1 ] 0.33(1) | [ 1 1 1 ] 0.26(2) | [ 0 1 1 ] 1.19(2) |

**Table S5.** Results of the crystal structure refinement of the chloride DP doped powder samples

**Тable S6.** Refined coordinates of atoms and their isotropic thermal parameters in chloride DP doped powder samples

| (Cl6) Cs_2_Ag_0.4_Na_0.6_In_0.9_Er_0.1_Cl_6_, Sp.Gr. *I*4*/mmm* | | | | | | |
| --- | --- | --- | --- | --- | --- | --- |
| Atom | Wyck off position | *x/a* | *y/b* | *z/c* | Occupation | В_iso._, Å^2^ |
| Cs | 4*d* | 0 | 1/2 | 1/4 | 1 | 1.54(8) |
| M1 | 2*b* | 0 | 0 | 1/2 | 0.4Ag+0.6Na | 1.2(3) |
| M2 | 2*a* | 0 | 0 | 0 | 0.9In+0.1 Er | 0.93(14) |
| Cl1 | 4*e* | 0 | 0 | 0.229(2) | 1 | 2.4(4) |
| Cl2 | 8*h* | 0.2611(14) | *x* | 1/2 | 1 | 1.2(2) |
| (Cl7) Cs_2_Ag_0.4_Na_0.6_In_0.9_Sb_0.09_Er_0.01_Cl_6_, Sp.Gr. $Fm\bar{3}m$ | | | | | | |
| Atom | Wyck off position | *x/a* | *y/b* | *z/c* | Occupation | В_iso._, Å^2^ |
| Cs | 8*c* | 1/4 | 1/4 | 1/4 | 1 | 1.52(10) |
| M1 | 4*b* | 1/2 | 1/2 | 1/2 | 0.4Ag+0.6Na | 3.5(4) |
| M2 | 4*d* | 0 | 0 | 0 | 0.9In+0.01 Er  +0.09 Sb | 0.3(2) |
| Cl | 24*e* | 0.2335(8) | 0 | 0 | 1 | 1.2(2) |
| (Cl8) Cs_2_Ag_0.4_Na_0.6_In_0.9_Sb_0.05_Er_0.05_Cl_6_, Sp.Gr. *I*4*/mmm* | | | | | | |
| Atom | Wyck off position | *x/a* | *y/b* | *z/c* | Occupation | В_iso._, Å^2^ |
| Cs | 4*d* | 0 | 1/2 | 1/4 | 1 | 1.93(7) |
| M1 | 2*b* | 0 | 0 | 1/2 | 0.4Ag+0.6Na | 1.5(2) |
| M2 | 2*a* | 0 | 0 | 0 | 0.9In+0.05 Er  +0.05 Sb | 1.25(12) |
| Cl1 | 4*e* | 0 | 0 | 0.228(2) | 1 | 3.4(4) |
| Cl2 | 8*h* | 0.2624(12) | *x* | 1/2 | 1 | 1.4(2) |

**Table S7.** Interatomic distances and coordination numbers (C.N.) of atoms in the chloride DP doped powder samples

| Atoms | Cl6 | Cl7 | Cl8 | C.N. |
| --- | --- | --- | --- | --- |
|  | Interatomic distances, Å | | |  |
| M1– 2Cl1  M1– 4Cl2 | 2.85(2)  2.749(10) | 2.806(9) | 2.86(2)  2.764(8) | 6 |
| M2– 2Cl1  M2– 4Cl2 | 2.41(2)  2.516(10) | 2.458(9) | 2.40(2)  2.503(8) | 6 |
| Cs– 4Cl1  Cs– 8Cl2 | 3.729(1)  3.72(2) | 3.7260(4) | 3.732(1)  3.72(2) | 12 |

**Table S8.** Batch and analytical compositions of the obtained powder samples

| Composition | Cs  (at.%) | Ag (at.%) | Na (at.%) | In  (at.%) | Sb  (at.%) | Er (at.%) | Cl  (at.%) |
| --- | --- | --- | --- | --- | --- | --- | --- |
| (Cl6) Cs_2_Ag_0.4_Na_0.6_In_0.9_Cl_6_ **0.1ErCl_3_** | 20.5 (20.0) | 3.7 (4.0) | 7.4  (6.0) | 10.3 (9.0) | 0 | 0.5  (1.0) | 57.6 (60.0) |
| (Cl7) Cs_2_Ag_0.4_Na_0.6_In_0.9_Cl_6_ 0.09 SbCl_3_ **0.01ErCl_3_** | 20.5 (20.0) | 4.5 (4.0) | 6.0  (6.0) | 9.5  (9.0) | 0.9  (0.9) | 0.2  (0.1) | 58.4 (60.0) |
| (Cl8) Cs_2_Ag_0.4_Na_0.6_In_0.9_Cl_6_ 0.05SbCl_3_ **0.05ErCl_3_** | 22.7  (20.0) | 3.5 (4.0) | 4.2 (6.0) | 10.0 (9.0) | 0.5  (0.5) | 0.2  (0.5) | 58.9 (60.0) |

The values in brackets are calculated from the batch compositions. The error of EDAX is within 0.2%.

**Table S9.** The crystallographic characteristics and the details of the refinement of the structures of the Cs_2_Ag_0.4_Na_0.6_InCl_6_,

Cs_2_Ag_0.4_Na_0.6_In_0.9_Cl_6_ 0.1ErCl_3_ (Cl6) and Cs_2_Ag_0.4_Na_0.6_In_0.9_Cl_6_ 0.09SbCl_3_ 0.01ErCl_3_ (Cl7) samples (single crystal XRD method)

| Single crystal emperical formula | **Cs_2_Ag_0.292_Na_0.708_InCl_6_** | **Cs_2_Ag_0.285_Na_0.715_In_0.971_Er_0.029_Cl_6_** | **Cs_2_Ag_0.160_Na_0.840_In_0.893_Er_0.017_Sb_0.090_Cl_6_** |
| --- | --- | --- | --- |
| Formula weight | 641.16 | 642.09 | 631.40 |
| S.G. | $Fm\bar{3}m$ (№225) | $Fm\bar{3}m$ (№225) | $Fm\bar{3}m$ (№225) |
| Unit cell parameters, *Å* | *a*=10.5008(3) | *a*=10.5018(2) | *a*=10.5148(2) |
| *V*, *Å ^3^* | 1157.89(10) | 1158.22(7) | 1162.53(7) |
| Number of atoms in the unit cell | 40 | 40 | 40 |
| *Z* | 4 | 4 | 4 |
| Calculated density, *g/sm^3^* | 3.678 | 3.682 | 3.608 |
| Absorbtion coefficient, *mm^-1^* | 10.064 | 10.205 | 9.912 |
| Radiation type,wavelength, *Å* | Mo Kα,  0.71073 | Mo Kα,  0.71073 | Mo Kα,  0.71073 |
| Single-crystal diffractometer | Bruker AXS X8 APEX II | Bruker AXS X8 APEX II | Bruker AXS X8 APEX II |
| *F*(000) | 1130 | 1131 | 1113 |
| *Θ* range for data collection | 3.881– 55.638 | 3.881– 55.700 | 3.876– 55.597 |
| Range of indexes | -24≤*h*≤24; -24≤*k*≤24  -24≤*l*≤24 | -24≤*h*≤24; -24≤*k*≤24  -24≤*l*≤24 | -24≤*h*≤24; -24≤*k*≤24  -24≤*l*≤24 |
| Reflections collected | 55180 | 53827 | 53835 |
| Independent reflections | 437 | 439 | 440 |
| Refinement method | Full-matrix least-square on *F*^2^ | Full-matrix least-square on *F*^2^ | Full-matrix least-square on *F*^2^ |
| Data/restraints/parameters | 437/0/9 | 439/0/10 | 440/0/10 |
| Goodness-of-fit on *F*^2^ | 1.045 | 1.060 | 1.043 |
| *R* [*I>2σ(I)*] | *R*1=0.0362  *wR*2=0.0833 | *R*1=0.0340  *wR*2=0.0870 | *R*1=0.0305  *wR*2=0.0775 |
| *R* (all data) | *R*1=0.0626,  *wR*2=0.0995 | *R*1=0.0582,  *wR*2=0.1009 | *R*1=0.0481,  *wR*2=0.0886 |
| Shape; colour | grown, natural faces;  transparent white | grown, natural faces;  transparent white | grown, natural faces;  transparent yellow |
| Crystal size, µm | 75×65×50 | 80×60×50 | 70×65×50 |
| Extinction coefficient | 0.0013(2) | 0.00092(19) | 0.0038(4) |
| The highest difference peak and deepest hole, *Å^-3^* | 1.334and-2.082 | 1.354 and -2.066 | 1.509 and -1.620 |

**Table S10.** Atomic coordinates in the structure of the single crystals Cs_2_Ag_0.292_Na_0.708_InCl_6_, Cs_2_Ag_0.285_Na_0.715_In_0.971_Er_0.029_Cl_6_, Cs_2_Ag_0.160_Na_0.840_In_0.893_Er_0.017_Sb_0.090_Cl_6_ (single crystal XRD method)

| Atom | Position | *x/a* | *y/b* | *z/c* | Occ. |
| --- | --- | --- | --- | --- | --- |
| **Cs_2_Ag_0.292_Na_0.708_InCl_6_** | | | | | |
| Cs  M1  In  Cl | 8*c*  4*b*  4*d*  24*e* | 1/4  1/2  0  0.23829(9) | 1/4  1/2  0  0 | 1/4  1/2  0  0 | 1  0.292(5)Ag +0.708(5)Na  1  1 |
| **Cs_2_Ag_0.285_Na_0.715_In_0.971_Er_0.029_Cl_6_** | | | | | |
| Cs  M1  M2  Cl | 8*c*  4*b*  4*d*  24*e* | 1/4  1/2  0  0.23831(10) | 1/4  1/2  0  0 | 1/4  1/2  0  0 | 1  0.285(6)Ag+0.715(6)Na  0.971(7)In+0.029(7)Er  1 |
| **Cs_2_Ag_0.160_Na_0.840_In_0.893_Er_0.017_Sb_0.090_Cl_6_** | | | | | |
| Cs  M1  M2  Cl | 8*c*  4*b*  4*d*  24*e* | 1/4  1/2  0  0.23823(9) | 1/4  1/2  0  0 | 1/4  1/2  0  0 | 1  0.160(7)Ag+0.840(7)Na  0.893(7)In+0.017(7)Er+0.090Sb  1 |

**Table S11.** Anisotropic parameters of the atoms in the structure of the single crystals Cs_2_Ag_0.292_Na_0.708_InCl_6_, Cs_2_Ag_0.285_Na_0.715_In_0.971_Er_0.029_Cl_6_, Cs_2_Ag_0.160_Na_0.840_In_0.893_Er_0.017_Sb_0.090_Cl_6_ (single crystal XRD method)

| Atom | *U_eq._, Å^2^* | *U*_11_ | *U*_22_ | *U*_33_ | *U*_23_ | *U*_13_ | *U*_12_ |
| --- | --- | --- | --- | --- | --- | --- | --- |
| **Cs_2_Ag_0.292_Na_0.708_InCl_6_** | | | | | | | |
| Cs  M1  In  Cl | 0.03022(15)  0.0228(4)  0.01593(14)  0.0305(3) | 0.03022(15)  0.0228(4)  0.01593(14)  0.0155(3) | 0.03022(15)  0.0228(4)  0.01593(14)  0.0380(4) | 0.03022(15)  0.0228(4)  0.01593(14)  0.0380(4) | 0  0  0  0 | 0  0  0  0 | 0  0  0  0 |
| **Cs_2_Ag_0.285_Na_0.715_In_0.971_Er_0.029_Cl_6_** | | | | | | | |
| Cs  M1  M2  Cl | 0.02973(19)  0.0230(5)  0.01613(14)  0.0304(3) | 0.02973(19)  0.0230(5)  0.01613(14)  0.0158(4) | 0.02973(19)  0.0230(5)  0.01613(14)  0.0377(4) | 0.02973(19)  0.0230(5)  0.01613(14)  0.0377(4) | 0  0  0  0 | 0  0  0  0 | 0  0  0  0 |
| **Cs_2_Ag_0.160_Na_0.840_In_0.893_Er_0.017_Sb_0.090_Cl_6_** | | | | | | | |
| Cs  M1  M2  Cl | 0.02998(18)  0.0239(6)  0.01587(12)  0.0308(2) | 0.02998(18)  0.0239(6)  0.01587(12)  0.0156(3) | 0.02998(18)  0.0239(6)  0.01587(12)  0.0384(3) | 0.02998(18)  0.0239(6)  0.01587(12)  0.0384(3) | 0  0  0  0 | 0  0  0  0 | 0  0  0  0 |

*U_eq_*_._ is defined as one third of the trace of the orthogonalized U_ij_ tensor. The anisotropic temperature factor exponent takes the form: -2*π*^2^[*h*^2^*a**^2^*U*_11_ + ... + 2*hka*b*U*_12_]

**Table S12.** Interatomic distances (d, Å) and coordination numbers (C.N.) of the atoms in the structure of the single crystals Cs_2_Ag_0.292_Na_0.708_InCl_6_, Cs_2_Ag_0.285_Na_0.715_In_0.971_Er_0.029_Cl_6_, Cs_2_Ag_0.160_Na_0.840_In_0.893_Er_0.017_Sb_0.090_Cl_6_ (single crystal XRD method)

| **Cs_2_Ag_0.292_Na_0.708_InCl_6_** | | | | | | | | | | |
| --- | --- | --- | --- | --- | --- | --- | --- | --- | --- | --- |
| Atoms | | | | d, Å | | | | | C.N. | |
| Cs | | –12Cl | | 3.71463(11) | | | | | 12 | |
| *M1 | | – 6Cl | | 2.7482(9) | | | | | 6 | |
| In | | – 6Cl | | 2.5022(9) | | | | | 6 | |
| **Cs_2_Ag_0.285_Na_0.715_In_0.971_Er_0.029_Cl_6_** | | | | | | **Cs_2_Ag_0.160_Na_0.840_In_0.893_Er_0.017_Sb_0.090_Cl_6_** | | | | |
| Atoms | | | d, Å | | C.N. | Atoms | | d, Å | | C.N. |
| Cs | –12Cl | | 3.71498(8) | | 12 | Cs | – 12Cl | 3.71960(8) | | 12 |
| **M1 | – 6Cl | | 2.7482(11) | | 6 | ***M1 | – 6Cl | 2.7525(9) | | 6 |
| **M2 | – 6Cl | | 2.5027(11) | | 6 | ***M2 | – 6Cl | 2.5049(9) | | 6 |

*M1= 29.2 % Ag+ 70.8 % Na

**M1= 28.5 % Ag+ 71.5 % Na; **M2 = 97.1 % In + 2.9 % Er

***M1= 16.0 % Ag+ 84.0 % Na; ***M2 = 89.3 % In + 1.7 % Er + 9.0 % Sb

**Table S13.** Batch and analytical compositions of the single crystals

| Composition of the sample | Cs  (at.%) | Ag (at.%) | Na (at.%) | In  (at.%) | Sb (at.%) | Er (at.%) | Cl  (at.%) |
| --- | --- | --- | --- | --- | --- | --- | --- |
| Cs_2_Ag_0.4_Na_0.6_InCl_6_ | 20.6  20.0^*^  20.0^**^ | 3.8  2.9^*^  4.0^**^ | 7.7  7.1^*^  6.0^**^ | 10.6  10.0^*^  10.0^**^ | 0 | 0 | 57.3  60.0^*^  60.0^**^ |
| Cl6  Cs_2_Ag_0.4_Na_0.6_In_0.9_Er_0.1_Cl_6_ | 20.3  20.0^*^  20.0^**^ | 2.6  2.8^*^  4.0^**^ | 7.1  7.2^*^  6.0^**^ | 10.8  9.7^*^  9.0^**^ | 0 | 0.1  0.3^*^  1.0^**^ | 59.1  60.0^*^  60.0^**^ |
| Cl7  Cs_2_Ag_0.4_Na_0.6_In_0.9_Sb_0.09_Er_0.01_Cl_6_ | 19.0  20.0^*^  20.0^**^ | 3.1  1.6^*^  4.0^**^ | 8.4  8.4^*^  6.0^**^ | 9.7  8.9^*^  9.0^**^ | 0.9  0.9^*^  0.9^**^ | 0.1  0.2^*^  0.1^**^ | 58.8  60.0^*^  60.0^**^ |

^*^The values obtained from crystal structure calculation (Tables S9-S12).

^**^The values are calculated from the batch compositions.

The error of EDS is within 0.2%.


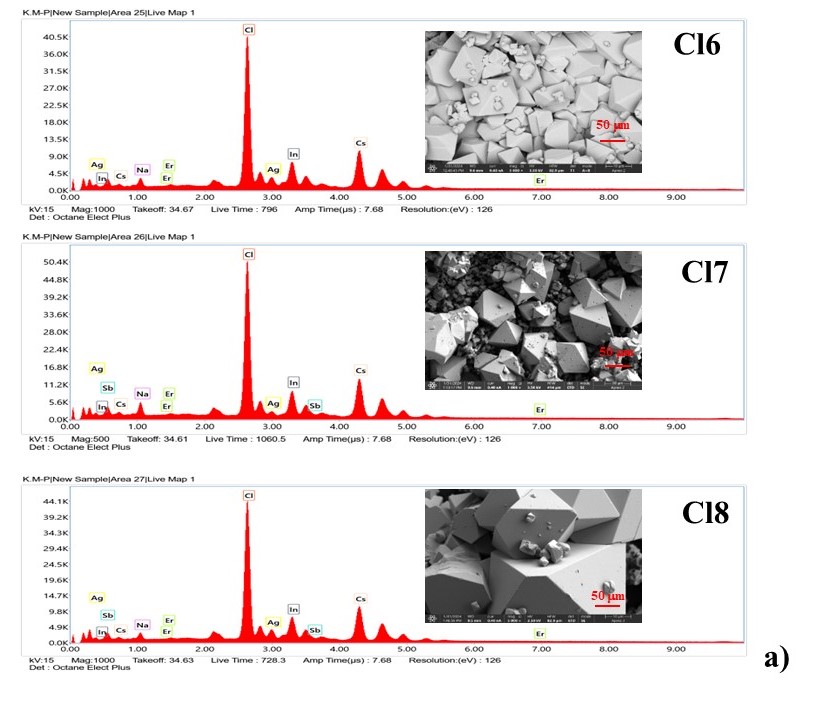


**Fig. S3** SEM photos of the Cl6, Cl7, Cl8 samples and their EDS results.


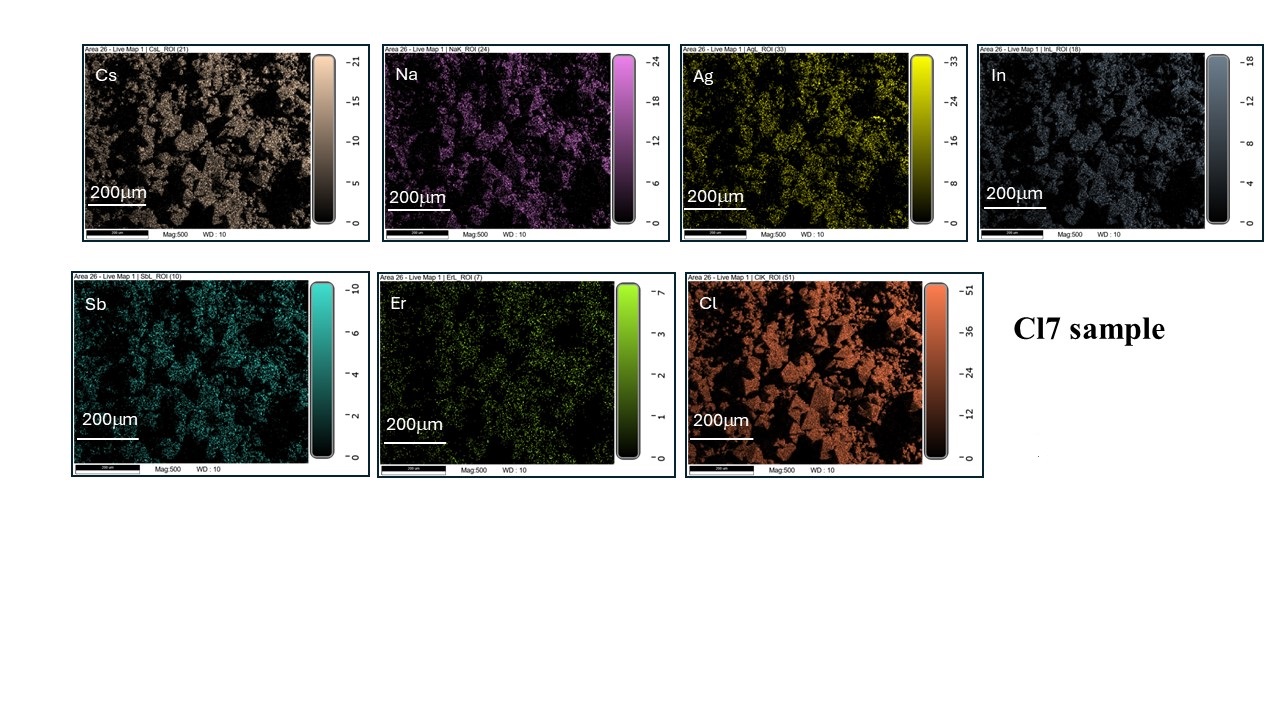


**Fig. S4** Maps of the elements of the Cs_2_Ag_0.4_Na_0.6_In_0.9_Sb_0.09_Er_0.01_Cl_6_ (Cl7) powder sample.


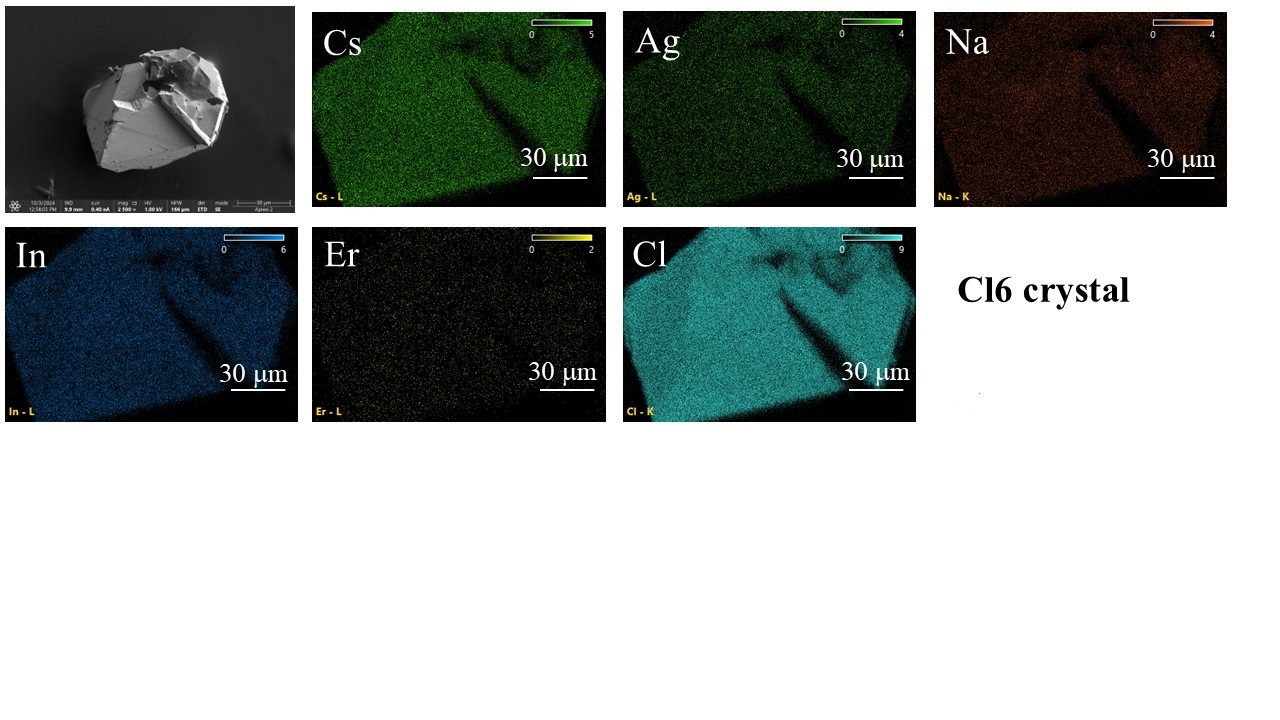


**Fig. S5** Maps of the elements of the Cl6 single crystal Cs_2_Ag_0.285_Na_0.715_In_0.971_Er_0.029_Cl_6_ (Cs_2_Ag_0.4_Na_0.6_In_0.9_Er_0.1_Cl_6_ batch composition).


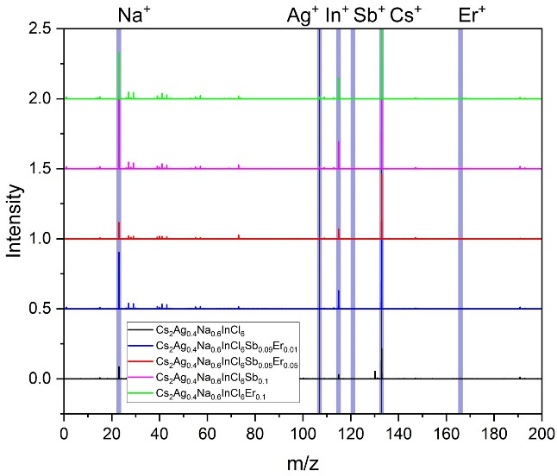


**Fig. S6.** ToF-SIMS mass spectra collected for Cs_2_Ag_0.4_Na_0.6_InCl_6_ (black line), Cs_2_Ag_0.4_Na_0.6_In_0.9_Sb_0.09_Er_0.01_Cl_6_ (Cl7) (blue line), Cs_2_Ag_0.4_Na_0.6_In_0.9_Sb_0.05_Er_0.05_Cl_6_ (Cl8) (orange line), Cs_2_Ag_0.4_Na_0.6_In_0.9_Sb_0.1_Cl_6_ (magenta line), Cs_2_Ag_0.4_Na_0.6_In_0.9_Er_0.1_Cl_6_ (Cl6) (green line). The area of the elements of interest are highlighted.


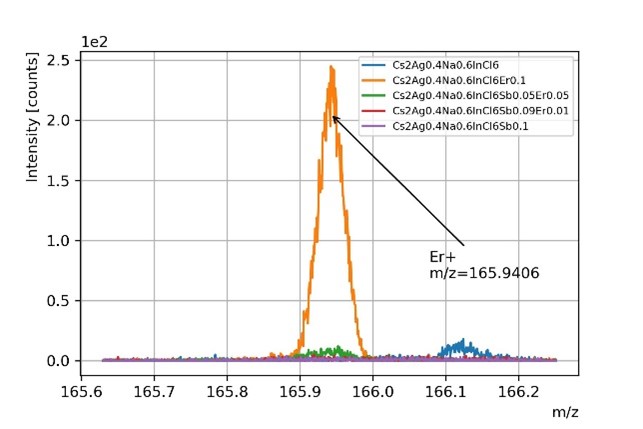


**Fig. S7.** Mass spectra from the area of Er^+^ signal plotted for all samples.


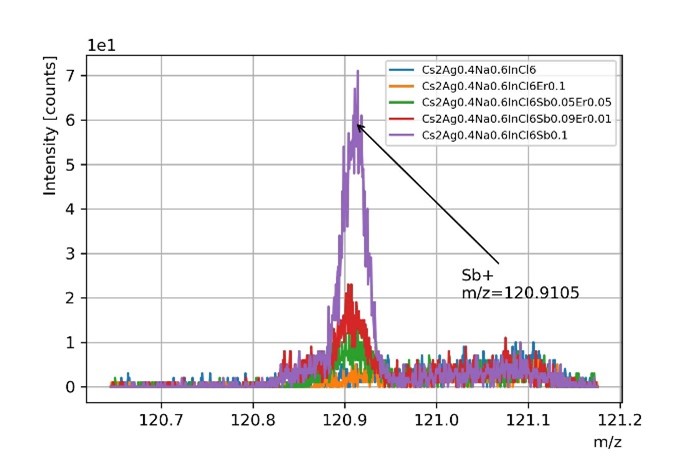


**Fig. S8.** Mass spectra from the area of Sb^+^ signal plotted for all samples.


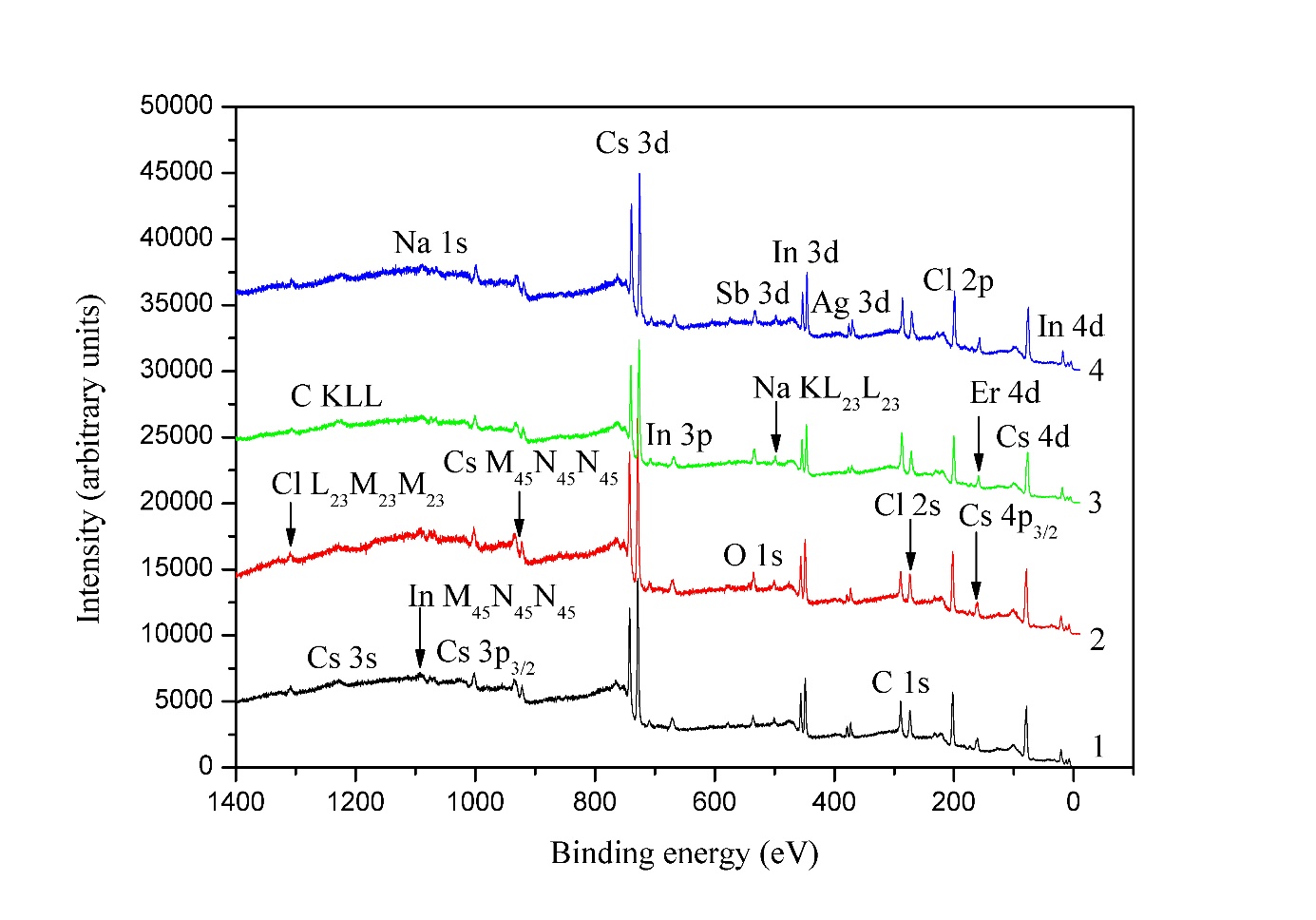


**Fig. S9.** Survey XPS spectra of (1) Cs_2_Ag_0.4_Na_0.6_InCl_6_, (2) Cs_2_Ag_0.4_Na_0.6_In_0.9_Sb_0.1_Cl_6_, (3) Cs_2_Ag_0.4_Na_0.6_In_0.9_Sb_0.09_Er_0.01_Cl_6_ (Cl7) and (4) Cs_2_Ag_0.4_Na_0.6_In_0.9_Sb_0.05_Er_0.05_Cl_6_ (Cl8) (Note: due to several XPS spectral features, only the most important ones are marked).


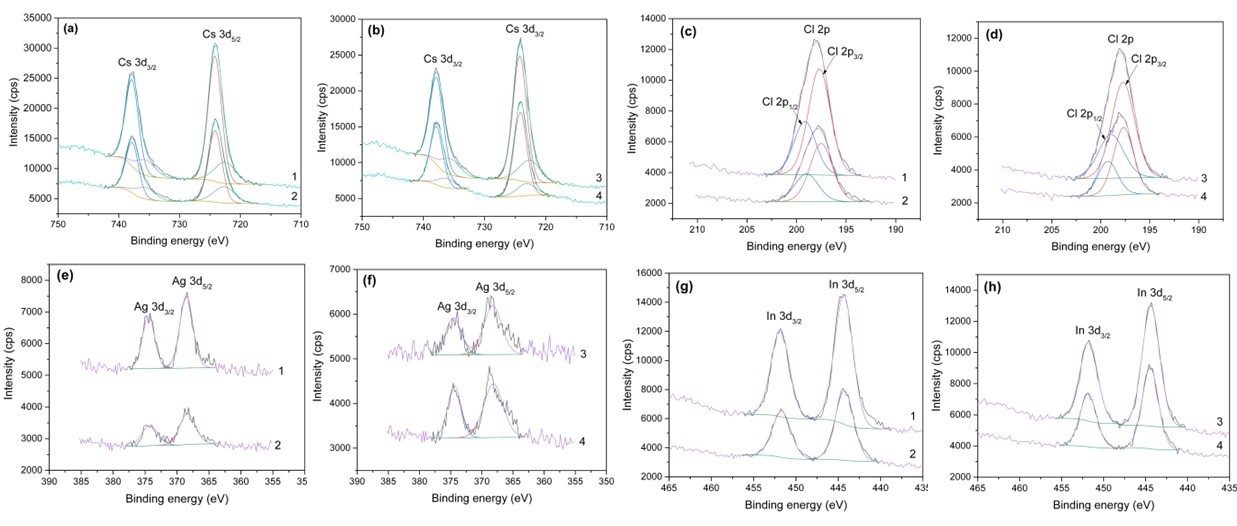


**Fig. S10.** XPS core-level spectra of (1) Cs_2_Ag_0.4_Na_0.6_InCl_6_, (2) Cs_2_Ag_0.4_Na_0.6_In_0.9_Sb_0.1_Cl_6_, (3) Cs_2_Ag_0.4_Na_0.6_In_0.9_Sb_0.09_Er_0.01_Cl_6_ (Cl7) and (4) Cs_2_Ag_0.4_Na_0.6_In_0.9_Sb_0.05_Er_0.05_Cl_6_ (Cl8): (a, b) Cs 3*d*, (c, d) Cl 2*p*, (e, f) Ag 3*d*, and (g, h) In 3*d* (Note: due to a number of XPS spectral features, only the most important ones are marked).

**Table S14.** Experimental (*E*_exp_) [41] and calculated^*^ (*E*_calc_) energy levels of a free Er^3+^ ion

| *^2S+1^L_J_* | *J* | *E*_exp_, cm^-1^ | *E*_calc_, cm^-1^ |
| --- | --- | --- | --- |
| ^4^I | 15/2  13/2  11/2  9/2 | 0  6480  10110  12350 | 0  6480  10062  12344 |
| ^4^F | 9/2 | 15180 | 16022 |
| ^4^S | 3/2 | 18290 | 19684 |
| ^2^H | 11/2 | - | 19857 |
| ^4^F | 7/2  5/2  3/2 | 20400  22070  22410 | 21607  23334  23616 |
| ^2^H | 9/2 | - | 24886 |
| ^2^K | 15/2 | - | 27773 |
| ^4^G | 11/2 | - | 27879 |
| ^2^G | 7/2 | - | 28868 |
| ^4^G | 9/2 | - | 29125 |
| ^2^D | 3/2 | - | 32874 |
| ^2^K | 13/2 | - | 33052 |
| ^4^G | 5/2  7/2 | - | 35330  35392 |
|  |  |  |  |

^*^The fitting parameters are *Z_eff_* = 17.191; *K_rel_* =24.728.

Reference:

RS1. L. Akselrud, Yu. Grin. *J. Appl. Crystallogr*. **2014**, *47*, 803
